# Taming the giant: towards a global sectional taxonomy for the big genus *Artemisia*

**DOI:** 10.1186/s12870-026-08951-7

**Published:** 2026-05-28

**Authors:** Bohan Jiao, Meng Wei, Guohao Niu, Xiyang Chen, Yifan Liu, Daniel M. Katumo, Jiahao Shen, Daniel Vitales, Joan Vallès, Filip Verloove, Andrey S. Erst, Alexey P. Seregin, Akiko Soejima, Florian Jabbour, Artem Leostrin, Wei Wang, Tiangang Gao

**Affiliations:** 1 State Key Laboratory of Plant Diversity and Specialty Crops, Institute of Botany, Chinese Academy of Sciences, Beijing, 100093 China; 2 China National Botanical Garden, Beijing, 100093 China; 3 University of Chinese Academy of Sciences, Beijing, 100049 China; 4 Institute of Botany, Jiangsu Province and Chinese Academy of Sciences, Nanjing, 210014 China; 5 Botanical Institute of Barcelona (IBB, CSIC-Ajuntament de Barcelona), Pg. del Migdia, s.n., Barcelona, 08038 Spain; 6 Laboratori de Botànica - Unitat Associada al CSIC - IRBio, Universitat de Barcelona, Av. Joan XXIII 27-31, Barcelona, Catalonia 08028 Spain; 7 Meise Botanic Garden, Nieuwelaan 38, Meise, B-1860 Belgium; 8 Central Siberian Botanical Garden SB RAS, Novosibirsk, Russia; 9 Faculty of Biology, M. V. Lomonosov Moscow State University, Moscow, 119991 Russia; 10 Faculty of Advanced Science and Technology, Kumamoto University, 2-39-1 Kurokami, Chuo-Ku, Kumamoto, 860-8555 Japan; 11 Institut de Systématique Evolution Biodiversité (ISYEB), Muséum National d’Histoire Naturelle, CNRS, Sorbonne Université, EPHE, Université Des Antilles, 57 Rue Cuvier CP39, Paris, 75005 France; 12 Herbarium (LE) of Komarov Botanical Institute, Russian Academy of Sciences, St. Petersburg, 197022 Russian Federation

**Keywords:** *Artemisia*, Asteraceae, Taxonomy, Infrageneric, Morphology, Phylogeny, Big genus

## Abstract

**Background:**

*Artemisia* is a big genus of significant medicinal, ecological, and economic importance. However, a global sectional taxonomy with complete morphological and nomenclatural details is still lacking despite decades of research.

**Results:**

Based on phylogenomic data (202 nuclear low copy genes plus two ribosomal DNA markers) covering 78% of accepted *Artemisia* species and morphological evidence (20 characters), we propose a global sectional taxonomy for the genus with morphological and nomenclatural details. This taxonomy accommodates 502 of the 505 accepted species (three remain unplaced owing to insufficient evidence) and recognizes 24 sections in eight subgenera, for which morphological descriptions, diagnostic keys, and nomenclatural acts are provided. Sixteen sections are newly established, and five are re-circumscribed.

**Conclusions:**

This comprehensive sectional taxonomy establishes a robust framework for the infrageneric classification of *Artemisia*. It resolves long‑standing taxonomic uncertainties and provides a foundation for diverse research—from understanding the evolution and ecology of the genus to guiding the sustainable use of its medicinally and ecologically important species. Moreover, it presents a methodological case for addressing taxonomic complexity in other big plant genera.

**Supplementary Information:**

The online version contains supplementary material available at 10.1186/s12870-026-08951-7.

## Introduction

**Fig. 1:**
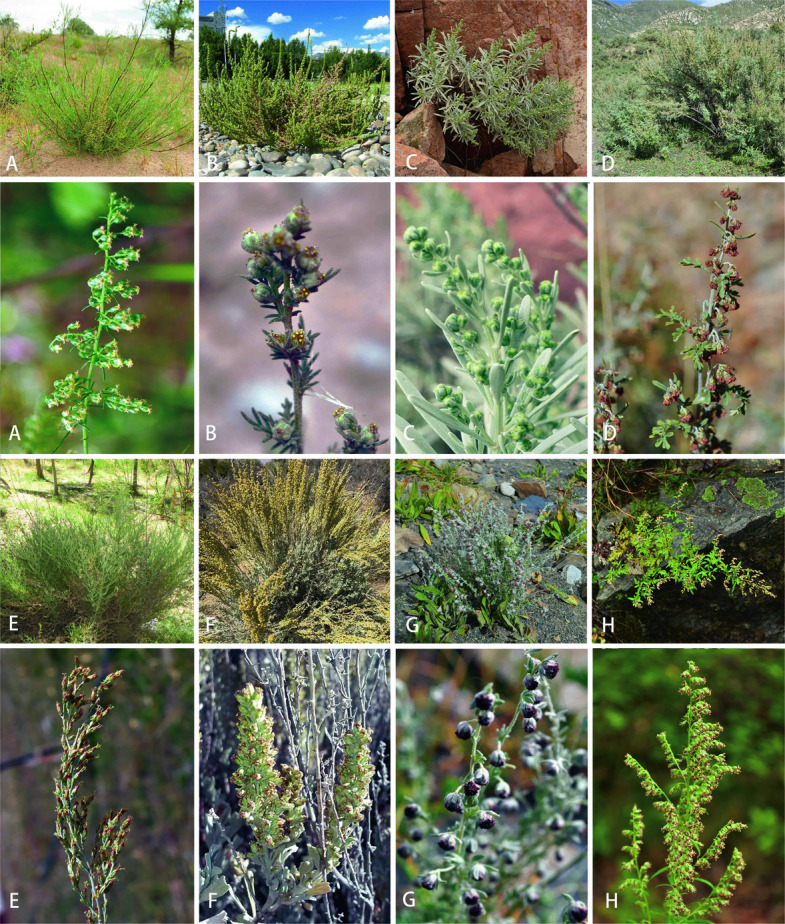
Representative species of *Artemisia*, showing the diversity of habitus (upper rows) and capitula (lower rows). **A** *A. ordosica* (subg. *Dracunculus*). **B** *A. pectinata* (subg. *Pectinatae*). **C** *A. chinensis* (subg. *Pacifica*). **D** *A. vestita* (subg. *Ponticae*). **E** *A. brevifolia* (subg. *Seriphidium*). **F** *A. tridentata* (subg. *Tridentatae*). **G** *A. frigida* (subg. *Absinthium*). **H** *A. qinlingensis* (subg. *Artemisia*)., **F** *A. tridentata* provided by © Kristin Sweeney, cited from https://www.inaturalist.org

*Artemisia* L., a big genus of the sunflower family (Asteraceae), comprises over 500 species mainly distributed across Northern Hemisphere [1–4]. The genus exhibits remarkable morphological and habitus diversity (Fig. 1) and holds significant medicinal, ecological and economic value. Many of its species are renowned for diverse uses: as medicinal plants (*A. annua*, *A. argyi*), forage for livestock (*A. tridentata*, *A. herba-alba*), culinary herbs (*A. dracunculus*), and key ingredients in beverages and ornamentals (*A. absinthium*, *A. arborescens*, *A. vulgaris*) [5]. A comprehensive and phylogenetically-informed infrageneric taxonomy is essential to guide the sustainable utilization of these valuable resources. Despite decades of effort, such a taxonomy remains lacking [6–10].

*Artemisia* was first described by Linnaeus [11]. The genus is generally characterized by two types of capitula (heterogamous-disciform or homogamous-discoid), spineless or shortly spined pollen (*Artemisia* pollen type [12]), and ribless cypselae [5, 13–15]. Nevertheless, none of these characters alone are diagnostic for *Artemisia* [4]. Morphologically, 13 monotypic or small genera, namely *Artemisiastrum* Rydb., *Artemisiella* Ghafoor, *Crossostephium* Less., *Elachanthemum* Y.Ling & Y.R.Ling, *Filifolium* Kitam., *Hippolytia* Poljakov, *Kaschgaria* Poljakov, *Mausolea* Poljakov, *Neopallasia* Poljakov, *Picrothamnus* Nutt., *Stilpnolepis* Krasch., *Sphaeromeria* Nutt. and *Turaniphytum* Poljakov, have long been recognized allied to *Artemisia* [4, 16–18]. The taxonomic status of these genera has been controversial for a long time. Early molecular phylogenetic studies based on a few DNA markers provided initial insights [9, 19–23], and more recently, phylogenomic evidence has strongly supported merging most of these genera with *Artemisia*, with the exception of *Artemisiella*, *Elachanthemum*, *Hippolytia*, and *Stilpnolepis* [1, 24]. Similarly, *Seriphidium* (Besser ex Hook.) Fourr., a big lineage with ca. 150 species, was historically segregated from *Artemisia* based on capitulum morphology [3, 13]. However, detailed morphological and molecular study [10], along with recent phylogenomic evidence [1, 10], have firmly established *Seriphidium* as a subgenus within *Artemisia*. In addition, *Ajaniopsis* Shih, a genus endemic to the Tibet Plateau, has also been merged into *Artemisia* with strong support [1, 25].

The controversy over the infrageneric taxonomy of *Artemisia* has persisted for a long time. As summarized in Table 1, the infrageneric taxonomy proposed since Linnaeus has varied in the geographical coverage and species involved. Early treatments (pre-Shultz [4]) relied primarily on morphological characters (e.g., capitulum type), while Shultz and subsequent taxonomists began to integrate molecular phylogenetic evidence alongside morphology [23]. Recently, we utilized single nucleotide polymorphisms (SNP) data from the nuclear genome to delineate the genus into eight strongly supported clades [24] (Table 1). Through expanded genomic sampling, broader species coverage, and the integration of comprehensive morphological data, we further resolved the phylogeny into eight clades and twenty‑four corresponding subclades [1]. Although these studies established a robust phylogenetic framework, key details—including the complete sectional taxonomy and supporting morphological descriptions, diagnostic keys, and nomenclatural acts—were not presented. Consequently, the resulting sectional taxonomy has remained informal and lacks the validated, stable reference needed for downstream application across disciplines.

**Table 1:** Comparison of different infrageneric taxonomies of *Artemisia*

Rank	Taxa								Reference	Geographical Coverage	Number of treated Species
Genus	*Artemisia*								Linnaeus (1753)	global	19
Sections	*Absinthium*	*Abrotanum*				*Seriphidium*		*Dracunculus*	Besser (1829, 1831, 1834, 1835);	global	173
									Candolle (1837); Ledebour (1844–46)	global	180
Sections	*Euartemisia*					*Seriphidium*		*Dracunculus*	Gray (1886)	North America	44
Subgenera	*Euartemisia*					*Seriphidium*		*Euartemisia*	Rouy (1903)	France	13
Subgenera	*Absinthium*	*Abrotanum*				*Seriphidium*		*Dracunculus*	Rydberg (1916)	North America	123
Sections						*Seriphidium*	*Tridentatae*				
Sections	*Absinthium*	*Abrotanum*				*Seriphidium*		*Dracunculus*	Hall and Clements (1923)	North America	29
Subgenera	*Artemisia*					*Seriphidium*		*Dracunculus*	Poljakov (1961a)	Soviet Union	181
Sections	*Artemisia*							*Dracunculus*	Tutin *&* *al.* (1976)	Europe	57
Subgenera	*Artemisia*					*Seriphidium*		*Dracunculus*	Podlech (1986)	Iran	64
Genera	*Artemisia*					*Seriphidium*		*Artemisia*	Ling (1980–90); Ling *&* *al.* (2011)	global	472
Subgenera	*Artemisia*					*Seriphidium*		*Dracunculus*			
Subgenera	*Absinthium*	*Artemisia*				*Seriphidium*	*Tridentatae*	*Dracunculus*	Shultz (2006)	North America	51
Subgenera	*Absinthium*	*Artemisia*			*Pacifica*	*Seriphidium*	*Tridentatae*	*Dracunculus*	Hobbs and Baldwin (2013)	global	112
Subgenera	*Absinthium*	*Artemisia*	*Ponticae*	*Pectinatae*	*Pacifica*	*Seriphidium*	*Tridentatae*	*Dracunculus*	Jiao & al. (2023)	global	205
Subgenera	*Absinthium*	*Artemisia*	*Ponticae*	*Pectinatae*	*Pacifica*	*Seriphidium*	*Tridentatae*	*Dracunculus*	Jiao & al. (2025)	global	505

In this work, we propose a complete sectional taxonomy of *Artemisia*, including formal descriptions and a diagnostic key based on a robust phylogenetic framework and integrated morphological data under the principles of monophyly and identifiability [26], thereby establishing the nomenclatural stability and details required for its consistent scientific use.

## Materials and methods

### Taxon sampling

Our sampling includes 394 species of *Artemisia*, covering all eight currently recognized subgenera [1, 24] including all recently merged genera (*Ajaniopsis*, *Crossostephium*, *Filifolium*, *Kaschgaria*, *Mausolea*, *Neopallasia*, *Picrothamnus*, *Sphaeromeria*, and *Turaniphytum*), as in Jiao et al. [1]. We also included 14 species from closely related genera (*Artemisiella*, *Chrysanthemum*, *Ajania*, *Phaeostigma*, *Elachanthemum*) as outgroups [19, 27]. All samples represent one individual per species and were identified by Drs. Bohan Jiao and Tiangang Gao (Institute of Botany, Chinese Academy of Sciences). Voucher specimens have been deposited in publicly accessible herbaria; detailed collection data, herbarium codes, and deposition numbers are provided in Supplementary Material 1 and acknowledged in the Acknowledgements section.

### Phylogenomic analyses

Of the 408 species analyzed, 310 species had genome-skimming data, from which, we generated 202 low-copy nuclear gene sequences and two nuclear ribosomal DNA sequences (ITS, ETS), the remaining 98 species had only nuclear ribosomal DNA data (ITS, ETS). A gigamatrix combines a supermatrix (many taxa, few genes) and a phylogenomic matrix (few taxa, many genes), balancing taxonomic coverage and genomic depth for comprehensive and robust phylogenetic inference [28]. Here, we constructed a concatenated gigamatrix comprsing 202 low-copy nuclear genes and two nuclear ribosomal DNA regions (ITS, ETS) (10.6084/m9.figshare.28164335). The phylogeny was reconstructed using IQ-TREE v.2.0.6 [29], based on the gigamatrix, treating each nuclear gene and ribosomal DNA markers as a separate partition. Substitution models were selected via the corrected Akaike information criterion (AICc) calculated using ModelFinder [30] in IQ-TREE.

### Morphological analyses

In total, twenty morphological characters were analyzed, comprising 13 macromorphological (Table 2) and seven micromorphological (Table 3). The macromorphological characters included: 1) life form, 2) plant height, 3) capitulum type, 4) synflorescence, 5) capitulum diameter, 6) leaf shape, 7) number of leaf segment pairs, 8) leaf size, 9) leaf area, 10) leaf length–width ratio, 11) leaf segment length, 12) leaf segment width, and 13) leaf type. For macromorphological characters, measurements were taken from three individuals per species using ImageJ software (https://imagej.nih.gov/ij/), and averagd, with cross-validation against published literature. Detailed macromorphological character states are presented in Table 2. The seven micromorphological characters examined were 1) corolla shape of disk floret, 2) corolla shape of marginal floret, 3) style morphology of disk floret, 4) style morphology of marginal floret, 5) shape of anther apical appendages, 6) shape of anther thecal base, and 7) shape of anther collar. Due to material limitations, micromorphological analysis was ultimately perfomed on a pruned phylogenetic tree comprising 200 *Artemisia* species encompassing all eight subgenera and 24 sections. Floret samples were fixed in FAA, ultrasonically cleaned, treated with 5% NaOH treatment, mounted in Hoyer's solution and examined under a Leica DM5000B microscope. Corolla, style, and anther characters were documented following the terminology of Roque et al. [31] and Grossi et al. [32]. Detailed states are presented in Table 3. The phylogenetic signals for all twenty characters were assessed using Blomberg’*K* [33] and Pagel’s *λ* [34] in R v3.6.1 [35].

**Table 2:** Macromorphological characters, states and their phylogenetic signals of *Artemisia* (^*^ *P* < 0.01)

**No**	**Character**	**Character states**	Pagel's λ	Blomberg's K
1	Life form	1) Annual herbs; 2) Perennial herbs; 3) Subshrub/shrub	0.7863^*^	0.0967^*^
2	Plant height	1) Low: < 50 cm; 2) Medium: 50 cm ≤ height < 120 cm; 3) High: ≧ 120 cm	0.5430^*^	0.0031^*^
3	Capitulum type	1) *Artemisia* type: heterogamous-disciform; 2) *Dracunculus* type: heterogamous-disciform with central floret male; 3) *Absinthium* type: heterogamous-disciform, receptacle pubescent; 4) *Seriphidium* type: homogamous-discoid	0.9325^*^	1.9951^*^
4	Synflorescence	1) Corymb; 2) Panicle; 3) Raceme or single	0.4436^*^	0.0054
5	Capitulum diameter	1) Small: < 2.75 mm; 2) Middle: 2.75 mm ≤ diameter < 7 mm; 3) Big: ≧ 7 mm	0.6731^*^	0.0073
6	Leaf shape	1) Entire/3-lobed; 2) 1-pinnate; 3) 2-pinnate; 4) 3-pinnate	0.6868^*^	0.0068
7	Number of leaf segment pairs	1) ≧ 4 pairs; 2) 3 pairs; 3) 1 ≤ pairs < 3; 4) entire leaf (0 pair)	0.7580^*^	0.0294^*^
8	Leaf size	1) Small: < 560 mm^2^; 2) Medium: 560 mm^2^ ≤ size < 3600 mm^2^; 3) Big: *≧* 3600 mm^2^	0.6849^*^	0.1223^*^
9	Leaf area	1) Small: < 130 mm^2^; 2) Medium: 130 mm^2^ ≤ size < 600 mm^2^; 3) Big: ≧ 600 mm^2^	0.8086^*^	1.1043^*^
10	Leaf length–width ratio	1) 0 < ratio ≤ 2.2; 2) 2.2 < ratio ≤ 5; 3) ratio > 5	0.4422^*^	0.0064
11	Leaf segment length	1) Short: < 12 mm; 2) Medium: 12 mm ≤ length < 40 mm; 3) Long: ≧ 40 mm	0.8302^*^	0.0156^*^
12	Leaf segment width	1) Wide: ≧ 3 mm; 2) Narrow: < 3 mm	0.7303^*^	1.0528^*^
13	Leaf type	1) Type 1: Trilobed small leaf; 2) Type 2: 1-pinnatisect multiple-lobed small leaf; 3) Type 3: 2-palmate medium leaf; 4) Type 4: 2-pinnatisect, multiple-lobed medium long leaf; 5) Type 5: 2-pinnatisect medium leaf; 6) Type 6: 2-pectinately pinnatisect medium leaf; 7) Type 7: Entire toothed medium leaf; 8) Type 8: 5-lobed large leaf; 9) Type 9: 1-pinnatisect ovate lobe big leaf; 10) Type 10: 1-pinnatisect broad-lobed large leaf; 11) Type 11: 2-pectinately pinnatisect large leaf	0.9999^*^	1.5015^*^

**Table 3:** Micromorphological characters, states and their phylogenetic signals of *Artemisia* (^*^ *P* < 0.01)

**No**	**Character**	**Character states**	**Pagel's* λ***	Blomberg's K
1	Corolla shape of disk floret	1) Cup-shaped tubular; 2) Tubular; 3) Campanulate tubular	0.9999^*^	0.9672^*^
2	Corolla shape of marginal floret	1) Filiform; 2) Narrow tubular; 3) Conical	0.9999^*^	3.2239^*^
3	Style morphology of disk floret	1) Undivided; 2) Bifid, apex truncate; 3) Bifid, apex acute	0.9999^*^	4.4106^*^
4	Style morphology of marginal floret	1) Apex acute; 2) Apex retuse	0.9999^*^	2.4985^*^
5	Shape of anther apical appendages	1) Attenuate; 2) Acute; 3) Oblong	0.7223^*^	0.8994^*^
6	Shape of anther thecal base	1) Obtuse; 2) Sagittate; 3) Tailed	0.4809^*^	0.0050
7	Shape of anther collar	1) Oblong; 2) Balusterform	0.5233^*^	0.0021

### Sectional taxonomy establishment

**Fig. 2:**
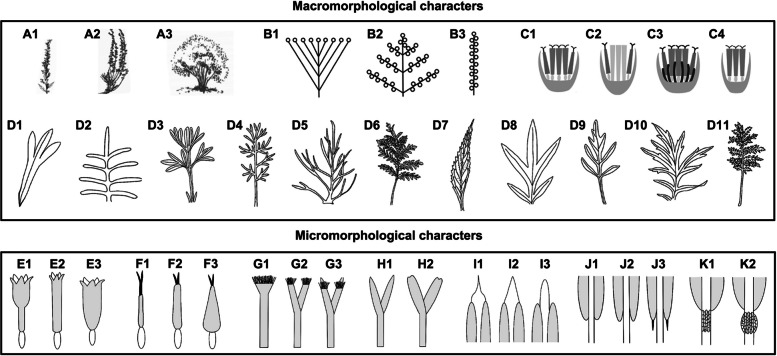
Morphological characters and their states of *Artemisia.* **A**-**D** Macromorphological characters. **A** Life form: A1. Annual herb; A2. Perennial herb; A3. Subshrub/shrub. **B** Synflorescence: B1. Corymb; B2. Panicle; B3. Raceme or single. **C** Capitulum type: C1. *Artemisia* type: heterogamous-disciform; C2. *Dracunculus* type: heterogamous-disciform with central floret male; C3. *Absinthium* type: heterogamous-disciform, receptacle pubescent; C4. *Seriphidium* type: homogamous-discoid. **D** Leaf type: D1. Type 1: Trilobed small leaf; D2. Type 2: 1-pinnatisect multiple-lobed small leaf; D3. Type 3: 2-palmate medium leaf; D4. Type 4: 2-pinnatisect, multiple-lobed medium long leaf; D5. Type 5: 2-pinnatisect medium leaf; D6. Type 6: 2-pectinately pinnatisect medium leaf; D7. Type 7: Entire toothed medium leaf; D8. Type 8: 5-lobed large leaf; D9. Type 9: 1-pinnatisect ovate lobe big leaf; D10. Type 10: 1-pinnatisect broad-lobed large leaf; D11. Type 11: 2-pectinately pinnatisect large leaf. **E**-**K** Micromorphological characters. **E** Corolla shape of disk floret: E1. Cup-shaped tubular; E2. Tubular; E3. Campanulate tubular. **F** Corolla shape of marginal floret: F1. Filiform; F2. Narrow tubular; F3. Conical. **G** Style morphology of disk floret: G1. Undivided; G2. Bifid, apex truncate; G3. Bifid, apex acute. **H** Style morphology of marginal floret: H1. Apex acute; H2. Apex retuse. **I** Shape of anther apical appendage: I1. Attenuate; I2. Acute; I3. Oblong. **J** Shape of anther thecal base: J1. Obtuse; J2. Sagittate; J3. Tailed. **K**. Shape of anther collar. K1. Oblong; K2. Balusterform

**Fig. 3:**
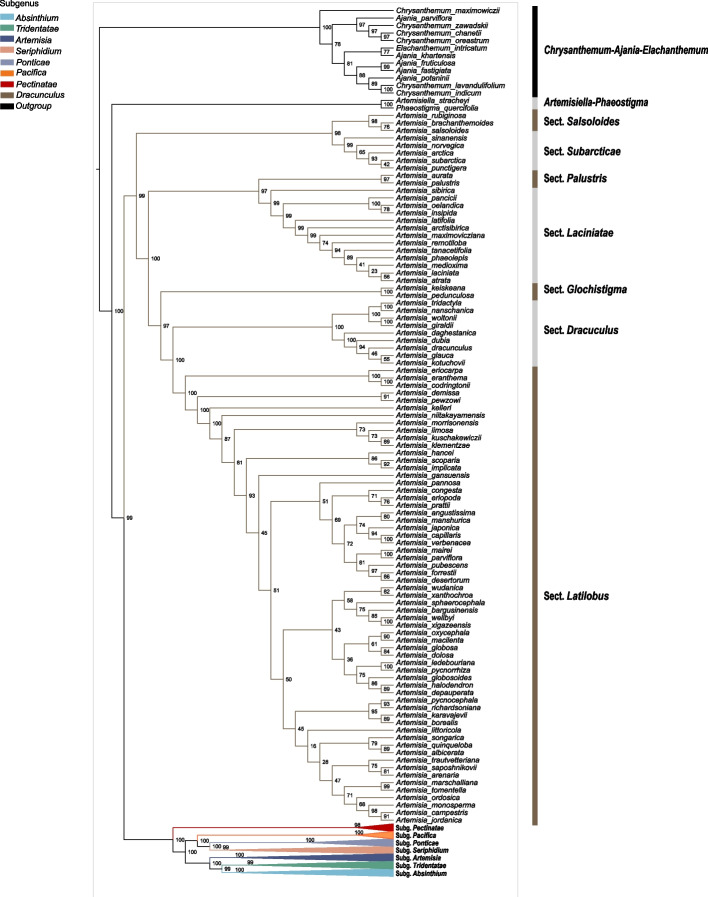
Phylogenetic relationships within *Artemisia* and allied genera, highlighting sections of *Artemisia* subg. *Dracunculus*. The maximum likelihood tree of 408 species (394 *Artemisia* + 14 allies) was inferred from a gigamatrix dataset comprising 202 low-copy nuclear genes and ITS/ETS sequences (Jiao & al., 2025). Branches are colored by subgenus, and node supports are indicated by bootstrap values from 100 replicates

**Fig. 4:**
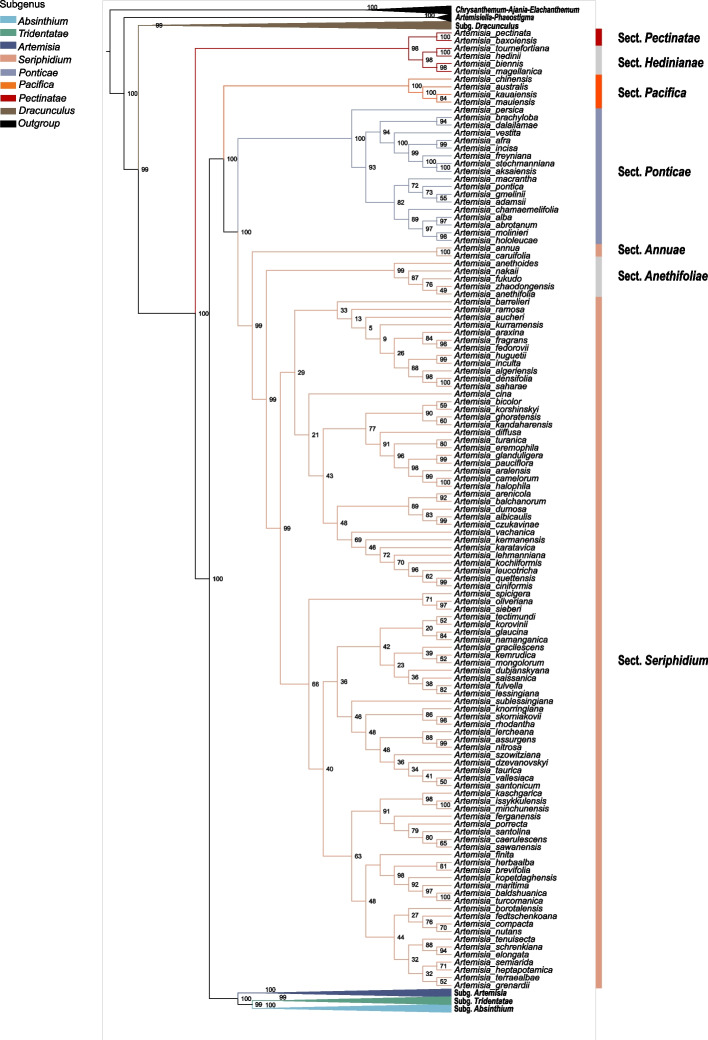
Phylogenetic relationships within *Artemisia* and allied genera, highlighting sections of *Artemisia* subg. *Pectinatae*, *Pacifica*, *Ponticae* and *Seriphidium*. The maximum likelihood tree of 408 species (394 *Artemisia* + 14 allies) was inferred from a gigamatrix dataset comprising 202 low-copy nuclear genes and ITS/ETS sequences (Jiao & al., 2025). Branches are colored by subgenus, and node supports are indicated by bootstrap values from 100 replicates

**Fig. 5:**
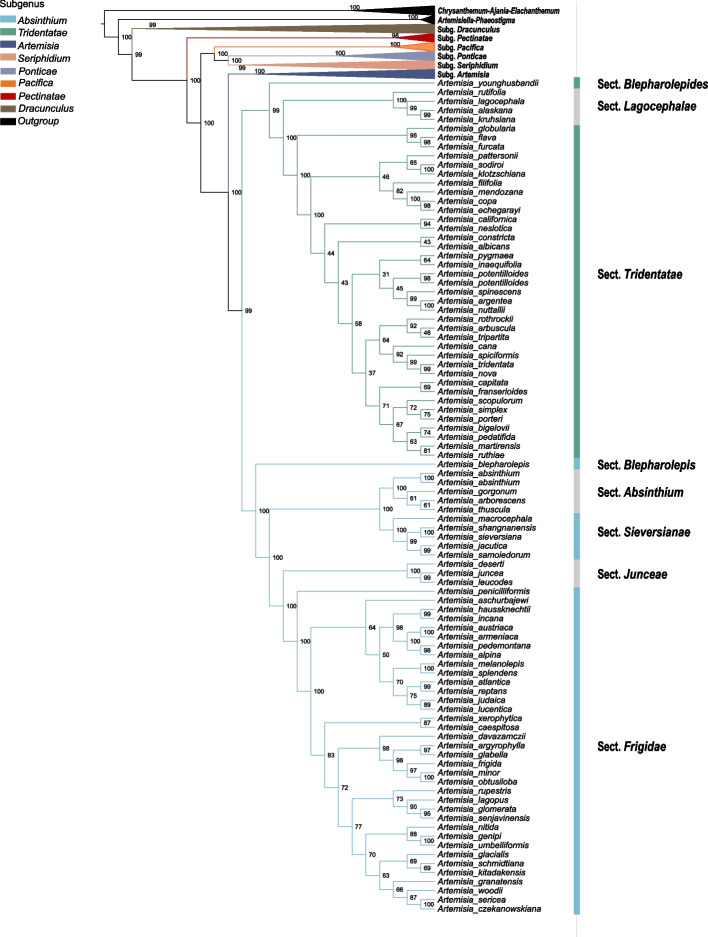
Phylogenetic relationships within *Artemisia* and allied genera, highlighting sections of *Artemisia* subg. *Tridentatae* and *Absinthium*. The maximum likelihood tree of 408 species (394 *Artemisia* + 14 allies) was inferred from a gigamatrix dataset comprising 202 low-copy nuclear genes and ITS/ETS sequences (Jiao & al., 2025). Branches are colored by subgenus, and node supports are indicated by bootstrap values from 100 replicates

**Fig. 6:**
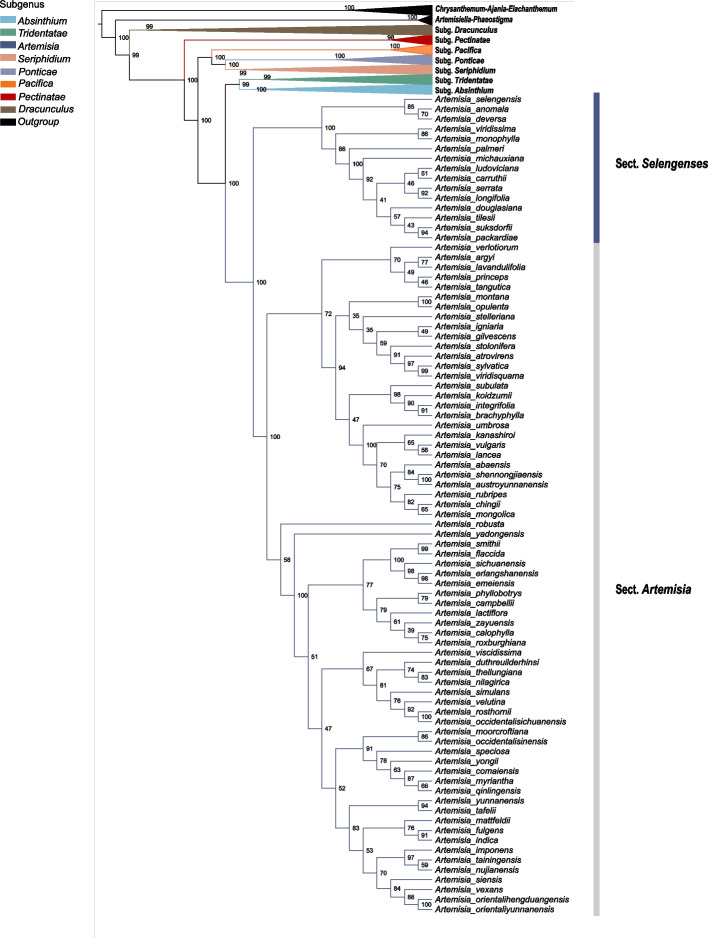
Phylogenetic relationships within *Artemisia* and allied genera, highlighting sections of *Artemisia* subg *Artemisia*. The maximum likelihood tree of 408 species (394 *Artemisia* + 14 allies) was inferred from a gigamatrix dataset comprising 202 low-copy nuclear genes and ITS/ETS sequences (Jiao & al., 2025). Branches are colored by subgenus, and node supports are indicated by bootstrap values from 100 replicates

Guided by the principles of monophyly and identifiability [36], we established a sectional taxonomy of *Artemisia* that recognizes eight subgenera and twenty-four sections. Accordingly, we selected the characters with high phylogenetic signals (*λ* ≧ 0.9 or *K* ≧ 1.5) and compiled a diagnostic key to all subgenera and sections, and formally defined each section with its accepted name, subgeneric placement, morphological characters and all the species inside (Supplementary Material 2). Definitions of the morphological characters, their states, and associated terminology for this new taxonomy are detailed in Tables 2 and 3, with some visually illustrated in Fig. 2. The accepted species names for *Artemisia* were primarily sourced from major databases, including Plants of the World Online (POWO), the Global Compositae Checklist (GCC), World Flora Online (WFO), and the Catalog of Life (CoL). This compilation was completed on 29 September 2024. In total, 502 of 505 accepted *Artemisia* species were classified into 8 subgenera and 24 sections, 394 species were assigned based on both molecular and morphological evidence, while 105 species solely on diagnostic morphological characters (Figs. 3, 4, 5 and 6, and Supplementary Material 1).

## Results and discussion

### Phylogeny and circumscription of *Artemisia*

We reconstructed a fully resolved phylogeny of *Artemisia* and its allied genera based on maximum likelihood (ML) analysis of a large nuclear matrix comprising 202 low-copy genes and two ribosomal DNA sequences. The tree included 394 *Artemisia* species (78% of the accepted species) and 14 outgroup species (Figs. 3, 4, 5 and 6; Supplementary Material 1). The results strongly support (BS = 100%) expanding the circumscription of *Artemisia* to include nine previously segregated genera—*Ajaniopsis*, *Crossostephium*, *Filifolium*, *Kaschgaria*, *Mausolea*, *Neopallasia*, *Picrothamnus*, *Sphaeromeria*, and *Turaniphytum* (Figs. 3, 4, 5 and 6) [19, 21, 22, 24, 25]. Now, the genus *Artemisia* is characterized by pollen with short spines or no spines (the so-called *Artemisia* pollen type [12]) and heterogamous-disciform (disc florets bisexual or functionally staminate, ray florets pistillate) or homogamous discoid (disc florets bisexual and fertile, ray florets absent) capitula. Achenes lacking ribs and pappus, along with paniculate synflorescences, provide additional diagnostic support. However, exceptions exist: pappose achenes occur in *A. chinensis* and *A. kauaiensis* [23]; ribbed achenes are known from *A. penicilliformis*, *A. cana*, *A. tridentata* and *A. umbelliformis* [25]; and racemose or corymbose synflorescences occasionally appear [1, 24].

### Infrageneric phylogeny of *Artemisia*

Based on the phylogenetic framework including 78% *Artemisia* species, we identified eight strongly supported clades and 24 highly supported subclades (Figs. 3, 4, 5 and 6; BS > 95%). We identified possible synapomorphies for these clades and subclades based on the morphological characters (Tables 2 and 3). All these robust clades and subclades are diagnosable by morphological characters (see the key below). We treated them as subgenera and sections respectively in the subsequent discussion. Among them, the earliest-diverging *A.* subg. *Dracunculus* contained the highest number subclades (seven) (Fig. 3). *A.* subg. *Absinthium* was resolved into five subclades, followed by *A.* subg. *Seriphidium* and *A.* subg. *Tridentatae*, each comprising three subclades (Figs. 4, and 5). *A.* subg. *Artemisia* and *A.* subg. *Pectinatae* were divided into two subclades (Figs. 4, and 6). In contrast, *A.* subg. *Pacificae* and *A.* subg. *Ponticae* remained undivided (Fig. 4).

### Taxonomic value of morphological characters

A total of twenty morphological characters were checked, including thirteen macromorphological (Table 2, Fig. 2A–D) and seven micromorphological characters (Table 3, Fig. 2E–K). The phylogenetic signals for all these characters were assessed. A strong signal is indicated when Pagel’s *λ* approaches 1 or Blomberg’s *K* exceeds 1 [33, 34]. Among these, eight characters showed strong phylogenetic signals (Pagel’s *λ* ≧ 0.9 or Blomberg’s *K* ≧ 1.5) [33, 34]: capitulum type, leaf type, leaf segment width, leaf area, as well as corolla shape and style morphology of both marginal and disk florets (Tables 2 and 3). These characters are therefore diagnostically valuable for delimiting subgenera and sections in *Artemisia*. The strong signal thresholds (Pagel’s *λ* ≧ 0.9 or Blomberg’s *K* ≧ 1.5) are used here as empirical guidelines, not strict cutoffs. For example, disk floret corolla shape shows *λ* = 0.9999 but *K* = 0.9672 (Table 3); one indicates strong phylogenetic signal, the other moderate [33, 34]. When the two do not both reach the high signal thresholds (i.e., only one is high), we evaluate its taxonomic value based on character distribution patterns across clades. For instance, campanulate-tubular corollas dominate in *Artemisia* subg. *Dracunculus*, *A*. subg. *Ponticae* and *A*. subg. *Absinthium*, whereas tubular corollas are typical of *A.* subg. *Pectinatae*, *A*. subg. *Pacifica*, *A*. subg. *Seriphidium*, and all three types occur in *A.* subg. *Tridentatae*. This character therefore remains taxonomically informative. We suggest that investigating more morphological characters in future work will improve the resolution of the infrageneric taxonomy of this highly complex genus.

### Synopsis of the subgenera and sections of the genus *Artemisia* L.

In the taxonomic treatment below, the subgenus and section assignments for all species are provided in Supplementary Material 2.

*Artemisia* L., Sp. Pl. 2: 845. 1753. – Type: *Artemisia vulgaris* L.

= *Oligosporus* Cass., Bull. Sci. Soc. Philom. Paris. 33. 1817. – Type: *Oligosporus campestris* (L.) Cass. (≡ *Artemisia campestris* L.).

= *Crossostephium* Less., Linnaea 6: 220. 1831. – Type: *Crossostephium chinense* (L.) Makino (≡ *Artemisia chinensis* L.).

= *Picrothamnus* Nutt., Trans. Amer. Philos. Soc. ser. 2, 7: 417. 1841. – Type: *Picrothamnus desertorum* Nutt. (≡ *Artemisia spinescens* D.C.Eaton).

= *Sphaeromeria* Nutt., Trans. Amer. Philos. Soc., n.s., 7: 401. 1841. – Type: *Sphaeromeria capitata* Nutt. (≡ *Artemisia capitata* (Nutt.) Sòn.Garcia, Garnatje, McArthur, Pellicer, S.C.Sand. & Vallès-Xirau).

= *Seriphidium* (Besser ex Less.) Fourr., Ann. Soc. Linn. Lyon, ser. 2, 17: 89. 1869. ≡ *Artemisia* subgen. *Seriphidium* Besser ex Less., Syn. Gen. Compos. 264. 1832. – Type: *Seriphidium maritimum* (L.) Poljakov (≡ *Artemisia marítima* L.).

= *Artemisiastrum* Rydb., N. Amer. Fl. 34(3): 285. 1916. – Type: *Artemisiastrum palmeri* (A.Gray) Rydb. (≡ *Artemisia palmeri* A.Gray).

= *Chamartemisia* Rydb., N. Amer. Fl. 34(3): 242. 1916. – Type: *Chamartemisia compacta* (H.M.Hall) Rydb. (≡ *Artemisia constricta* Sòn.Garcia, Garnatje, McArthur, Pellicer, S.C.Sand. & Vallès-Xirau ≡ *Sphaeromeria compacta* (H.M.Hall) A.H.Holmgren, L.M.Shultz & Lowrey ≡ *Tanacetum compactum* H.M.Hall).

= *Vesicarpa* Rydb., N. Amer. Fl. 34(3): 242. 1916. – Type: *Vesicarpa potentilloides* (A.Gray) Rydb. (≡ *Artemisia potentilloides* A.Gray).

= *Filifolium* Kitam., Acta Phytotax. Geobot. 9: 157. 1940. – Type: *Filifolium sibiricum* (L.) Kitam. (≡ *Artemisia sibirica* (L.) Maxim.).

= *Neopallasia* Poljakov, Not. Syst. Herb. Inst. Blot. Acad. Sc. URSS. 17: 429. 1955. – Type: *Neopallasia pectinata* (Pall.) Poljakov (≡ *Artemisia pectinata* Pall.).

= *Kaschgaria* Poljakov, Not. Syst. Herb. Inst. Bot. Ac. Sc. URSS 18: 282. 1957. – Type: *Kaschgaria brachanthemoicdes* (C.Winkl.) Poljakov (≡ *Artemisia brachanthemoides* C.Winkl.).

= *Mausolea* Poljakov, Trudy Inst. Bot. Akad. Nauk Kazakhsk. S. S. R. 11: 170. 1961. – Type: *Mausolea eriocarpa* (Bunge) Poljakov (≡ *Artemisia eriocarpa* Bunge).

= *Turaniphytum* Poljakov, Fl. URSS 26: 632, 880. 1961. – Type: *Turaniphytum eranthemum* (Bunge) Poljakov (≡ *Artemisia eranthema* Bunge).

= *Ajaniopsis* Shih, Acta Phytotax. Sin. 16(2): 87. 1978. – Type: *Ajaniopsis penicilliformis* Shih (≡ *Artemisia penicilliformis* (Shih) M.Wei & T.G.Gao).

Key to the subgenera and sections of *Artemisia*

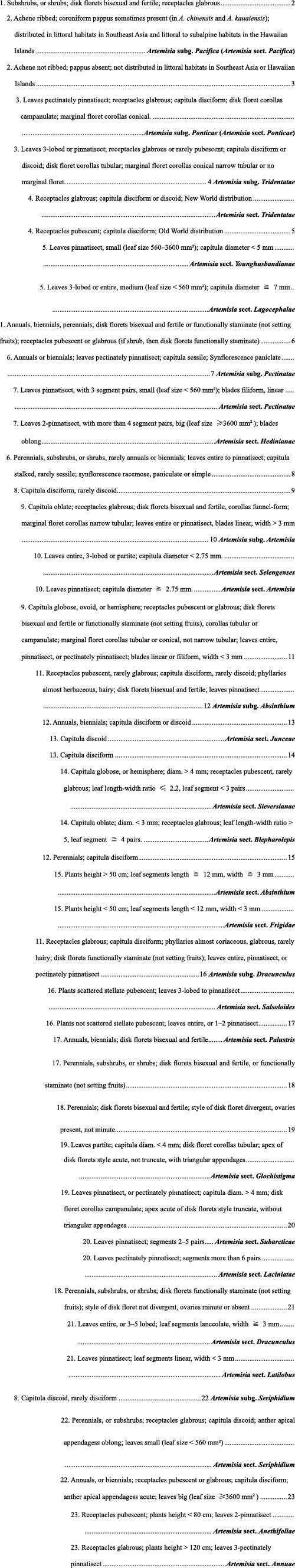



**1** ***Artemisia*** subg. ***Dracunculus*** (Besser) Rydb., N. Amer. Fl. 34(3): 251. 1916. – Type: *A. dracunculus* L. (Fig. 3). ≡ *Artemisia* sect. *Dracunculus* Besser, Bull. Soc. Imp. Naturalistes Moscou 8: 3, 8. 1835. – Type: *Artemisia dracunculus* L. = *Oligosporus* Cass., Bull. Sci. Soc. Philom. Paris. 33. 1817. – Type: *Oligosporus campestris* (L.) Cass. (≡ *Artemisia campestris* L.)

#### Description

Perennials or subshrubs, rarely annuals or biennials; 10–150 cm high; fibrous rooted or taprooted, caudices woody, perennial herbaceous species with rhizomes. Stems erect, rarely procumbent. Leaves deciduous, usually cauline, sometimes basal. Leaf morphology includes Types 1, 5, 7, 8, 10, 11. Synflorescence usually in panicle, sometimes in raceme or corymb. Capitula of *Dracunculus* or *Artemisia* type. Achenes without crown and ribs.

#### Species number and distribution

121 species; mainly distributed in temperate regions of Eurasia, a few extending to Northern America and Northern Africa.

#### Notes

*Artemisia* subg. *Dracunculus* was traditionally defined by a single key character: functionally staminate (non‑fruit‑setting) disk florets. Molecular phylogenetic analyses, however, reveal that this historically circumscribed subgenus is not monophyletic [9, 37]. With the exception of *A. filifolia* (which is nested within *A.* subg. *Tridentatae*), all the other species of the previous *A.* subg. *Dracunculus* clustered together with some species of *A.* subg. *Artemisia*. They formed the first diverging clade of the genus *Artemisia*. In the present revision, we expand *A.* subg. *Dracunculus* to include perennial species with pectinately pinnatisect large leaves (e.g., *A. latifolia*, *A. tanacetifolia*), annual species with pinnatisect leaves (e.g., *A. aurata*, *A. palustris*), *A. keiskeana*, as well as previously segregated genera such as *Filifolium* and *Kaschgaria*. Ecologically, most species occur in semi-arid grasslands, with some adapted to arid deserts (e.g., *A. eriocarpa*, *A. eranthema*) or forests (e.g., *A. angustissima*, *A. japonica*). Morphologically, the subgenus exhibits considerable variation in life form, leaf shape, and size, such that no single character reliably circumscribes it. Diagnosis therefore relies on combinations of morphological characters. A comprehensive morphological analysis remains essential for a deeper understanding of this big subgenus.

##### 1.1 *Artemisia* sect. *Palustris*

 B.H.Jiao & T.G.Gao, **sect.** **nov.** – Type: *A. palustris* L.

#### Description

Annual herbs; 30–70 cm high. Stems single, erect, glabrous. Middle stem leaves Type 5: 2- or 3-pinnatisect, segments 2–5 pairs, lobules narrowly linear-lanceolate or filiform. Synflorescence in broad panicle. Capitula of *Artemisia* type, sessile, usually 2–3 mm in diam.; disk florets bisexual and fertile. Disk floret corollas campanulate tubular; marginal floret corollas narrow tubular; style of disk florets bifid, apex truncate; style of marginal florets apex acute; anther apical appendages acute; anther thecal base sagittate; anther collar oblong.

#### Species number and distribution

2 species; Northeast China, Far East Russia, Mongolia, Korean Peninsula and Japan.

#### Notes

*Artemisia* sect. *Palustris* comprises two species, *A. aurata* and *A. palustris.* Phylogenetic analysis supports the transfer of this section from *A.* subg. *Artemisia* to *A.* subg. *Dracunculus* [1]. This section is characterized by an annual or biennial habit, globose capitula, yellow corollas, and large, Type 5 leaves. Ecologically, *A. palustris* occurs in steppe or forest-steppe habitats, whereas *A. aurata* prefers moist, rocky slopes. The circumscription of *Artemisia* sect. *Palustris* is equivalent to that of *A.* ser. *Auratae* Poljakov. However, according to Art. 40.1 and 40.3 of the ICN [38], *A.* ser. *Auratae* was not validly published, as it included more than one species in its 1961 protologue without a designated type [17]. We therefore formally propose the new sectional name here. 

##### 1.2 *Artemisia* sect. *Salsoloides*

Leonova. Novosti Sist. Vyssh. Rast. 25: 144. 1988. – Type: *A.* *salsoloides* Willd.

#### Description

Subshrub, 30–70 cm high; with thick, woody root, developing short, woody, strongly branched, perennial sterile shoots, scattered stellate pubescent. Leaves Type 1: small, no more than 3 cm long, middle steam leaves 3–5 lobes or entire. Synflorescence racemose or corymbose. Capitula *Artemisia* or *Dracunculus* type, sessile, usually 2–3 mm in diam.; disk florets bisexual and fertile (*Artemisia brachanthemoides*, *A. rubiginosa*) or functionally staminate (*A. salsoloides*). Disk floret corollas tubular; marginal floret corollas narrow tubular; style of disk florets bifid, apex truncate; style of marginal florets apex acute; anther apical appendages oblong; anther thecal base obtuse; anther collar balusterform or oblong.

#### Species number and distribution

3 species; Central and Eastern European part of Russia, Kazakhstan, North Caucasus, Northwest China (Xinjiang), and western Mongolia.

#### Notes

*Artemisia* sect. *Salsoloides* includes *A. salsoloides* and two former *Kaschgaria* species of (*Artemisia brachanthemoides* ≡ *Kaschgaria brachanthemoides*, *A. rubiginosa* ≡ *Kaschgaria komarovii*)*.* It is characterized by a racemose or corymbose synflorescence, but its species differ in capitulum type: *A. salsoloides* has *Dracunculus*-type capitula with functionally staminate disk florets, whereas the two former *Kaschgaria* species possess *Artemisia*-type capitula. Monophyly of the section and the relationships among its species are supported by ITS + ETS phylogeny [9]. Notably, species in this section have considerably larger genomes than most other *Artemisia*. The 2 C value of *A. salsoloides* is 11.40 pg [39] and that of *A. brachanthemoides* is 14.09 pg [40], compared to an average of about 6 pg for most species of *A.* subg. *Dracunculus* [39].

The name *Artemisia rubiginosa* was not validly published originally, as its protologue lacked a full and direct reference to the basionym [24]. According to ICN Art. 41.5 [38], it is therefore an illegitimate name. We validate it here as follows:

***Artemisia*** ***rubiginosa*** B.H.Jiao & T.G.Gao, **nom.** **nov.**

Replaced synonym: *Kaschgaria komarovii* (Krasch. & Rubtzov) Poljakov, Bot. Mater. Gerb. Bot. Inst. Komarova Akad. Nauk S.S.S.R. 18: 284. 1957.

Type:—Central Asia, Northwestern Mongolia, in arid steppes between Jamatei and Baityk-Bogdo, 6 August 1898, *E. Klementz 87* (LE!, LE01018058).

##### 1.3 *Artemisia* sect. *Subarcticae*

 B.H.Jiao & T.G.Gao, **sect.** **nov.** – Type: *A. subarctica* Krasch. = *Artemisia* [unranked] *Norvegica* Rydb., N. Amer. Fl. 34(3): 261. 1916 – Type: *A. norvegica* Fries.

#### Description

Short perennial herbs, 20–60 cm high. Stems few or single, 25–40 cm high, erect. Leaves Type 10 or 11: medium, pinnatisect or palmatisect. Synflorescence racemose. Capitula of *Artemisia* type, hemispherical, 5–12 mm in diam.; disk florets numerous (50–70). Disk floret corollas cup shaped tubular; marginal floret corollas narrow tubular; style of disk florets bifid, apex truncate; style of marginal florets apex retuse; anther apical appendages acute or attenuate; anther thecal base sagittate or obtuse; anther collar balusterform or oblong.

#### Species number and distribution

5 species; Arctic region.

#### Notes

All species of *A.* sect. *Subarcticae* have bisexual and fertile disk florets and glabrous receptacles, and were historically placed in *A.* subg. *Artemisia* [3, 17]. Diagnostic characters include large, oblate capitula (diam. > 5 mm) and narrow leaf segments (width < 3 mm), versus the smaller, oblong capitula (diam. < 4 mm) and broader leaf segments (width > 3 mm) in *A.* subg. *Artemisia*. The name *A.* ser. *Sinanenses* (as “Sinanenses”) was published by Ling [41] without a description or diagnosis, and is therefore not validly published according to ICN Art. 44.1 [38].

##### 1.4 *Artemisia* sect. *Laciniatae*

 (Kitam.) B.H.Jiao & T.G.Gao, **stat.** **nov.** ≡ *Artemisia* ser. *Laciniatae* Kitam., Act. Phytotax. Geobot. 8: 65. 1939. – Type: *A. laciniata* Willd. = *Artemisia* ser. *Latifoliae* Krasch., Mat. Hist. Fl. Veg. 2: 124. 1946. – Type: *A. latifolia* Ledeb.

#### Description

Perennial herbs; 50–70 cm high; rootstock horizontally creeping to obliquely rising. Leaves Type 11: Basal leaves long petiolate; middle stem leaves petiole 3–12 mm; 2-pectinately pinnatisect; segments 6–8 pairs; lobules toothed lanceolate; sometimes Type 5. Synflorescence in raceme, narrowly panicle or corymb (e.g. *A. sibirica* ≡ *Filifolium sibiricum*). Capitula of *Artemisia* type, hemispherical or oblate, 4–8 mm in diam. Disk floret corollas campanulate tubular; marginal floret corollas narrow tubular; style of disk florets bifid, apex truncate; style of marginal florets apex retuse; anther apical appendages acute or attenuate; anther thecal base sagittate, tailed or obtuse; anther collar oblong or balusterform.

#### Species number and distribution

16 species; Siberia and the Far East of Russia, the Korean Peninsula, Japan, and northern China.

#### Notes

*A.* sect. *Laciniatae* shares the same capitulum characters with *A.* sect. *Subarcticae*, and its species were also historically placed in *A.* subg. *Artemisia*. It can be distinguished from that subgenus by a narrowly paniculate or racemose synflorescence (vs. broadly paniculate) and by leaves that are 2-pectinately pinnatisect (vs. 1–2-pinnatisect). Within the section, *A. sibirica* (≡ *Filifolium sibiricum*) exhibits a somewhat distinct morphology (corymbose vs. racemose or narrowly paniculate synflorescence; Type 5 vs. Type 11 leaves), yet all molecular phylogenetic analyses to date consistently resolve it within this section.

##### 1.5 *Artemisia* sect. *Glochistigma*

 (Kitam.) B.H.Jiao & T.G.Gao, **stat.** **nov.** ≡ *Artemisia* subsect. *Glochistigma* Kitam., Act. Phytotax. Geobot. 8: 63. 1939. ≡ *Artemisia* ser. *Paniculigerae* Poljakov, Fl. URSS 26: 488. 1961. – Type: *A. keiskeana* Miq.

#### Description

Perennial, 50–100 cm high. Rhizome strong, with underground shoots. Leaves Type 7: leaf blades obovate or broadly cuneate, base attenuate, margin acutely serrate from middle to apex, apex rounded; green above, usually glabrous, pale green beneath, weakly hairy. Synflorescence paniculate. Capitula of *Artemisia* type, subglobose, 3–3.5 mm in diam.; style arms of disk florets not truncate, with triangular appendages. Disk floret corollas tubular; marginal floret corollas narrow tubular; style of disk florets bifid, apex acute; style of marginal florets apex retuse; anther apical appendages attenuate; anther thecal base sagittate; anther collar oblong.

#### Species number and distribution

2 species; Northeast China, Eastern Russia, Korean Peninsula, and Japan.

#### Notes

*Artemisia* sect. *Glochistigma* is characterized by *Artemisia*-type capitula and Type 7 leaves. It differs from *A.* subg. *Artemisia* in its globose (vs. oblong) capitula. A distinctive micro-morphological character is present in *A. keiskeana*, whose disk-floret style-branch apices are triangular—unlike the truncate apices typical of other members of *Artemisia*.

##### 1.6 *Artemisia* sect. *Dracunculus*

 Besser, Bull. Soc. Imp. Naturalistes Moscou 8: 3, 8. 1835. – Type: *A. dracunculus* L. = *Artemisia* [unranked] *Dracunculoides* Rydb., N. Amer. Fl. 34(3): 251. 1916. – Type: *A. dracunculoides* Pursh = *Artemisia* ser. *Subdigitatae* Krasch., Mat. Hist. Fl. Veg. 2: 176.1946. – Type: *A. subdigitata *Mattf. = *Artemisia* ser. *Phaeocephalae* Krasch., Mat. Hist. Fl. Veg. 2: 182.1946. – Type: *A. nanschanica *Krasch.

#### Description

Perennials, subshrubs or shrubs; 30–150 cm high. Perennial species with branched, woody rhizome. Leaves usually Type 1 (small, entire or 3–5 lobed); sometimes Type 8 (large, 5-lobed to pinnatipartite, *Artemisia dubia*). Synflorescence paniclate. Capitula of *Dracunculus* type, globose, 3–3.5 mm in diam. Disk floret corollas campanulate tubular; marginal floret corollas conical; style of disk florets undivided; style of marginal florets apex acute; anther apical appendages acute or attenuate; anther thecal base sagittate, tailed or obtuse; anther collar oblong or balusterform.

#### Species number and distribution

11 species; temperate regions of Eurasia and North America. *Artemisia dracunculus* and *A. dubia* are widely distributed, while the rest are narrowly distributed.

#### Notes

*Artemisia* sect. *Dracunculus* and *A.* sect. *Latilobus* form the core of *A.* subg. *Dracunculus*. Both sections share the defining character of *Dracunculus*-type capitula. They are distinguished primarily by life form and leaf morphology: sect. *Dracunculus* comprises mostly perennial herbs (with only two subshrub exceptions) that bear branched, woody rhizomes, and its leaf segments are wider than 2 mm. In contrast, all species of sect. *Latilobus* are shrubs with leaf segments less than 1.5 mm wide.

##### 1.7 *Artemisia* sect. *Latilobus*

 Y.R.Ling. Act. Phytotax. Sin. 18(4): 512. 1980. – Type: *A. japonica* Thunb. = *Artemisia* ser. *Pubescentes* Krasch., Mat. Hist. Fl. Veg. 2: 154. 1946. – Type: *A. pubescens *Ledeb. = *Artemisia* ser. *Scopariae* Krasch., Mat. Hist. Fl. Veg. 2: 156. 1946. – Type: *A. scoparia* Waldst. & Kit. = *Artemisia* ser. *Sphaerocephalae* Krasch., Mat. Hist. Fl. Veg. 2: 166.1946. – Type: *A. sphaerocephala* Krasch. = *Artemisia* ser. *Japonicae* Krasch., Mat. Hist. Fl. Veg. 2: 173.1946. – Type: *A. japonica* Thunb.

#### Description

Annuals, biennials, perennials, subshrubs or shrubs; 10–150 cm high. Perennial species with branched, woody rhizome. Leaves mostly Type 5 (2-pinnatisect medium leaves, with lobes linear or filiform). Synflorescence paniclate. Capitula of *Dracunculus* type, globose, 1.5–3 mm in diam. Disk floret corollas campanulate tubular; marginal floret corollas conical; style of disk florets undivided; style of marginal florets apex acute; anther apical appendages acute or attenuate; anther thecal base sagittate, tailed or obtuse; anther collar oblong or balusterform.

#### Species number and distribution

82 species; temperate regions of Eurasia and North America.

#### Notes

The expanded *A.* sect. *Latilobus* exhibits the greatest morphological diversity within *A.* subg. *Dracunculus*. Its species are characterized by *Dracunculus* type capitula, linear or filiform leaf segments on the middle stem, and (in most cases) the absence of a rhizome.

##### 2 *Artemisia* subg. *Pectinatae*

 B.H.Jiao & T.G.Gao, Ann. Bot. 131: 879. 2023. – Type: *A. pectinata* Pall (Fig. 4).

#### Description

Annual or biennial herbs; 12–40 cm or more than 100 cm high. Root vertical. Stems most often few or solitary, erect. Leaves Type 2 (small, pinnatisect multiple-lobed in sect. *Pectinatae*) or Type 11 (medium to large, pectinately pinnatisect in sect. *Hedinianae*). Synflorescence of short axillary spikes grouped into a slender, leafy panicle. Capitula of *Artemisia* type, glabrous. Achenes without crown and ribs.

#### Species number and distribution

*Artemisia* subg. *Pectinatae* comprises 10 species classified into two sections, and is distributed in temperate regions of Europe, Asia, Africa, North America, and South America. Most species grow in semi-arid grassland at low to medium altitudes, a few species such as *A. hedinii* and *A. baxoinensis* mainly grow in Qinghai-Tibet Plateau.

#### Note

*A.* subg. *Pectinatae* is characterized by annual or biennial herbs with pectinately pinnatisect leaves, and capitula arranged in dense narrow panicles. It was separated from *A.* subg. *Artemisia* by Jiao et al. [24]. Phylogenetic placement conflicts between nuclear and plastid data are likely attributable to chloroplast capture [24]. The densely clustered capitula confer a high reproductive capacity; for instance, the widespread weed *A. biennis* produces 400,000–1 million achenes per plant annually [42], a character that may facilitate its broad continental distribution. Several species were not included in our molecular sampling: *A. abyssinica* (Arabian Peninsula), *A. schimperi* and *A. tilhoana* (North and Central Africa), and *A. klotzschiana* (Mexico). They are provisionally assigned to *A.* subg. *Pectinatae*, pending future molecular phylogenetic study.

##### 2.1 *Artemisia* sect. *Pectinatae*

 B.H.Jiao & T.G.Gao, **sect.** **nov.** – Type: *A. pectinata* Pall.

#### Description

Annual or biennial herbs; 12–40 cm high. Root vertical. Stems most often few or solitary, erect. Leaves Type 2 (small, pinnatisect multiple-lobed). Synflorescence of short axillary spikes grouped into a slender, leafy panicle. Capitula of *Artemisia* type, globose or ovoid. Disk floret corollas cup shaped tubular; marginal floret corollas filiform or narrow tubular; style of disk florets bifid, apex truncate; style of marginal florets apex retuse; anther apical appendages acute or attenuate; anther thecal base sagittate or obtuse; anther collar oblong.

#### Species number and distribution

6 species; Northwest China, Mongolia, Central Asia, and East Siberia.

#### Notes

The two sections of *A.* subg. *Pectinatae* are distinguished by leaf length: *A.* sect. *Pectinatae* has leaves shorter than 2 cm, whereas *A.* sect. *Hedinianae* possesses leaves longer than 5 cm.

##### 2.2 *Artemisia* sect. *Hedinianae*

 (Y.R.Ling) B.H.Jiao & T.G.Gao, **stat.** **nov.** ≡ *Artemisia* ser. *Hedinianae* Y.R.Ling, Bull. Bot. Res., 8 (4): 17. 1988. – Type: *Artemisia hedinii* Ostenf. = *Artemisia* ser. *Tournefortianae* Y.R.Ling, Bull. Bot. Res., 8 (4): 18. 1988. – Type: *A. tournefortiana* Rchb.

#### Description

Annual or biennial herbs; more than 100 cm high or sometime 15–40 cm (*A. hedinii*). Root vertical. Stems most often few or solitary, erect. Leaves Type 11 (medium to large, pectinately pinnatisect). Synflorescence of short axillary spikes grouped into a slender, leafy panicle. Capitula of *Artemisia* type, globose. Disk floret corollas cup shaped tubular; marginal floret corollas narrow tubular; style of disk florets bifid, apex truncate; style of marginal florets apex acute; anther apical appendages acute or attenuate; anther thecal base sagittate; anther collar oblong.

#### Species number and distribution

4 species with a highly disjunct distribution, occurring in Central Asia, North America, and South America.

#### Notes

*Artemisia* ser. *Hedinianae* was originally established by Ling [43] to include only *A. hedinii*. Here, we raise this series to sectional rank as *A.* sect. *Hedinianae* and expand its circumscription to encompass *A.* ser. *Tournefortianae* as well as two additional species, *A. biennis* and *A. magellanica*.

##### 3 *Artemisia* subg. *Pacifica*

 C.R.Hobbs & B.G.Baldwin, J. Biogeogr. 40: 451. 2013. – Type: *A. australis *Less (Fig. 4).

#### Description

Small shrubs, branches sometimes trailing; 50–100 cm high. Leaves Type 9, medium to big, pinnatisect lobe ovate; leaves clustered near tips. Synflorescence paniculate or racemose. Capitula of *Artemisia* type, globose. Achene conspicuously 5-ribbed, glandular; pappus sometimes present (in *A. chinensis* and *A. kauaiensis*), teeth irregular. Disk floret corollas cup shaped tubular; marginal floret corollas filiform; style of disk florets bifid, apex truncate; style of marginal florets apex retuse; anther apical appendages acute; anther thecal base obtuse; anther collar oblong or balusterform.

#### Species number and distribution

4 species; one species (*A. chinensis*) of littoral habitats in Southeast Asia and three species (*A. australis*, *A. kauaiensis*, and *A. mauiensis*) of littoral to subalpine habitats in the Hawaiian Islands.

#### Notes

We recognize *A.* subg. *Pacifica* following the circumscription of Hobbs and Baldwin [23], without modification. It is characterized by a shrub habit, leaves clustered near the stem tips, and conspicuously 5-ribbed achenes. It includes *A. chinensis* (formerly treated as *Crossostephium chinense*), which, together with three Hawaiian endemics forms, a well-supported clade in molecular phylogenies [23].

##### 3.1 *Artemisia* sect. *Pacifica*

 (C.R.Hobbs & B.G.Baldwin) B.H.Jiao & T.G.Gao, **stat.** **nov.** ≡ *Artemisia *subg. *Pacifica* C.R.Hobbs & B.G.Baldwin, J. Biogeogr. 40: 451. 2013. – Type: *A. australis *Less.

The description and distribution of this section correspond to those of *A.* subg. *Pacifica* (see above).

##### 4 *Artemisia* subg. *Ponticae*

 (Rydb.) B.H.Jiao & T.G.Gao, Ann. Bot. 131: 879. 2023. ≡ *Artemisia* [unranked] *Ponticae* Rydb., N. Amer. Fl. 34(3): 280. 1916. ≡ *Artemisia* ser. *Ponticae* (Rydb.) Poljakov, Fl. URSS 26: 457. 1961. – Type: *A. pontica* L. (Fig. 4).

#### Description

Subshrubs or shrubs, 60–150 cm high; usually strongly aromatic. Root vertical. Stems numerous, erect. Leaves Type 5 (2-pinnatisect medium leaves, with lobes linear or filiform) or Type 6 (2-pectinately medium pinnatisect, with lobes lanceolate). Synflorescence paniclate. Capitula of *Artemisia* type, disciform. Achenes without crown and ribs. Disk floret corollas campanulate tubular; marginal floret corollas narrow tubular; style of disk florets bifid, apex truncate; style of marginal florets apex retuse; anther apical appendages acute or oblong; anther thecal base sagittate, tailed or obtuse; anther collar oblong or balusterform.

#### Species number and distribution

23 species; widely distributed in temperate regions of Eurasia, with a few species spreading to Africa (e.g. *A. afra*); growing mainly in semi-arid rocky hillsides, with a few species (e.g. *A. molinieri*) in wetlands.

#### Notes

This recently established subgenus comprises members transferred from *A.* sect. *Abrotanum* sensu Ling et al. [3]. Molecular phylogenetic analyses indicated that *A.* sect. *Abrotanum* was polyphyletic [23, 44]. Our phylogenomic data further resolved its subshrub and shrub species as a distinct clade, recognized here as *A.* subg. *Ponticae*. Its perennial herbaceous species (e.g., *A. tanacetifolia*) were placed in *A.* subg. *Dracunculus*, while annuals were assigned to *A.* subg. *Pectinatae* (e.g., *A. biennis*) and *A.* subg. *Seriphidium* (e.g., *A. annua*) [1]. Similar to *A.* subg. *Pectinatae*, nuclear and chloroplast phylogenies of *A.* subg. *Ponticae* show strongly discordant topologies (see Jiao et al. [1], Figs. 3, and 4). In the chloroplast tree, the species form two clades that correspond geographically to Europe and East Asia, respectively—mirroring the two subclades recovered in the nuclear phylogeny. This cytonuclear discordance may reflect historical hybridization. Currently, no consistent morphological characters distinguish these two subclades; thus, further subdivision into sections is unwarranted without additional morphological study.

##### 4.1 *Artemisia* sect. *Ponticae*

(Rydb.) B.H.Jiao & T.G.Gao, **stat.** **nov.** ≡ *Artemisia* [unranked] *Ponticae* Rydb., N. Amer. Fl. 34(3): 280. 1916. ≡ *Artemisia* ser. *Ponticae* (Rydb.) Poljakov, Fl. URSS 26: 457. 1961. ≡ *Artemisia* subg. *Ponticae* (Rydb.) B.H.Jiao & T.G.Gao, Ann. Bot. 131: 879. 2023. – Type: *A. pontica* L. = *Artemisia* sect. *Abrotanum* Bess., Bull. Soc. Nat. Mosc. 1 (8): 222. 1829. – Type: *A. abrotanum* L. = *Artemisia* ser. *Pectinatae* Kitam. Act. Phytotax. Geobot. 8: 65. 1939. – Type: *A. sacrorum* Ledeb*.* = *Artemisia* ser. *Persicae* Poljakov, Fl. URSS 26: 507. 1961. – Type: *A. persica* Boiss. 

Morphology, species number and distribution of *Artemisia* sect. *Ponticae* is the same as *Artemisia* subg. *Ponticae* presented above.

##### 5 *Artemisia* subg. *Seriphidium*

 Besser ex Less., Syn. Gen. Compos.: 264. 1832. – **Type**: *A. maritima* L. (Fig. 4).

#### Description

Annuals, biennials, subshrubs or shrubs, 30–150 cm high; strongly aromatic. Root vertical. Annuals or biennials, stems few (sect. *Anethifoliae*) or single (sect. *Annuae*); subshrubs or shrubs stem numerous (sect. *Seriphidium*), erect. Leaves Type 4 (small to medium, 2-pinnatisect, multiple-lobed, sect. *Seriphidium*), Type 5 (2-pinnatisect medium leaves, with lobes linear or filiform, sect. *Anethifoliae*), Type 6 (2-pectinately medium pinnatisect, with lobes lanceolate, sect. *Annuae*). Synflorescence paniclate. Capitula of *Artemisia*, *Absinthium* or *Seriphidium* type: disciform (sect. *Anethifoliae*, *Annuae*) or discoid (sect. *Seriphidium*); receptacles glabrous (sect. *Seriphidium*, *Annuae*) or pubescent (sect. *Anethifoliae*); disk florets bisexual and fertile. Achenes without crown and ribs.

#### Species number and distribution

128 species, Eurasia and North Africa.

#### Notes

The recircumscribed *A.* subg. *Seriphidium* comprises three morphologically distinct subclades. The first and largest corresponds to the traditional *A.* subg. *Seriphidium* [10] and is recognized here as *A.* sect. *Seriphidium*; it is characterized by *Seriphidium*-type capitula (discoid, lacking female florets) and forms the core of the subgenus with 119 species. The second, newly raised to sectional rank as *A.* sect. *Anethifoliae*, is defined by an annual or biennial habit and *Absinthium*-type capitula. The third, newly established as *A.* sect. *Annuae*, is distinguished by *Artemisia*-type capitula and large, 2–3-pectinately pinnatisect leaves (Type 6). Morphologically the most diverse lineage within *Artemisia*, *A.* subg. *Seriphidium* is distributed primarily across arid Central Asia, with most species exhibiting narrow endemism. A comprehensive taxonomic revision of this complex group remains an important priority.

##### 5.1 *Artemisia* sect. *Annuae*

 (Rydb.) B.H.Jiao & T.G.Gao, **stat.** **nov.** ≡ *Artemisia *[unrank] *Annuae* Rydb., N. Amer. Fl. 34 (3): 247. 1916. – Type: *A. annua* L.

#### Description

Annuals or biennials; 70–160 (200) cm high, usually strongly aromatic. Root vertical. Stems usually single, erect. Leaves Type 6 (medium, 2-pectinately pinnatisect, with lobes lanceolate). Synflorescence paniclate. Capitula of *Artemisia* type; corolla dark yellow. Disk floret corollas tubular; marginal floret corollas filiform; style of disk florets bifid, apex truncate; style of marginal florets apex retuse; anther apical appendages acute; anther thecal base tailed; anther collar oblong.

#### Species number and distribution

3 species; *Artemisia annua* in Europe and Asia, *A. caruifolia* and *A. calcicola* in East Asia (Southeast China, North India, Japan, Korea, Myanmar, Nepal, North Vietnam).

#### Notes

*Artemisia* sect. *Annuae* is sister to the remainder of *A.* subg. *Seriphidium* [1]. Although its species possess *Artemisia*-type capitula and were originally placed in *A.* subg. *Artemisia* sensu Ling et al. [3], they are distinguished by an annual or biennial life form, Type 6 leaves, and globose capitula, in contrast to the perennial habit, Type 7 or 10 leaves, and oblong capitula that characterize *A.* subg. *Artemisia*.

##### 5.2 *Artemisia* sect. *Anethifoliae*

 (Poljakov) B.H.Jiao & T.G.Gao, **stat.** **nov.** ≡ *Artemisia *ser.* Anethifoliae* Poljakov Fl. USSR 26: 522. 1961. – Type: *A. anethifolia *Weber ex Stechm.

#### Description

Annuals or biennials; 20–90 cm high, strongly aromatic. Root vertical. Stems few, erect. Leaves Type 5 (medium, 2-pinnatisect medium leaves, with lobes linear or filiform). Synflorescence paniclate. Capitula of *Absinthium* type, obovoid, nodding; corolla dark yellow. Disk floret corollas tubular; marginal floret corollas conical; style of disk florets bifid, apex truncate; style of marginal florets apex retuse; anther apical appendages acute; anther thecal base sagittate or tailed; anther collar oblong.

#### Species number and distribution

6 species; Northeast Asia; growing mainly in the saline-alkali soil of coastal areas or wastelands.

#### Notes

*Artemisia* sect. *Anethifoliae* is characterized by medium‑sized leaves (> 5 cm long) with linear segments longer than 5 mm. Most species in this section possess pubescent receptacles.

##### 5.3 *Artemisia* sect. *Seriphidium*

 (Besser ex Less.) Hooker, Fl. Bor.-Amer. (Hooker) 1(6): 325. 1833. – Type: *A. maritima *L. = *Artemisia* sect. *Pycnanthum* Filatova, Novosti Sist. Vyssh. Rast. 23: 236. 1986. – Type: *A. rhodantha *Rupr. = *Artemisia* sect. *Halophilum* Filatova, Novosti Sist. Vyssh. Rast. 23: 227. 1986. – Type: *A. halophila* = *Artemisia* sect. *Sclerophyllum* Filatova, Novosti Sist. Vyssh. Rast. 23: 224. 1986. – Type: *A. cina *Berg ex Poljak. = *Artemisia* sect. *Leucophyton* Filatova, Novosti Sist. Vyssh. Rast. 23: 222. 1986. – Type: *A. sieberi *Bess. = *Artemisia* sect. *Calciphilum* Filatova, Novosti Sist. Vyssh. Rast. 23: 217. 1986. – Type: *A. saharae *Pomel.

#### Description

Perennials, subshrubs or shrubs; 30–60 cm high; strongly aromatic. Root vertical, with numerous short fertile branches. Stem numerous, erect. Leaves Type 4 (small to medium, 2-pinnatisect, multiple-lobed). Synflorescence paniculate or racemose. Capitula of *Seriphidium* type, oblong. Disk floret corollas cup shaped tubular; marginal floret corollas absent; style of disk florets bifid, apex truncate; style of marginal florets absent; anther apical appendages oblong; anther thecal base sagittate or tailed; anther collar oblong.

#### Species number and distribution

119 species; Eurasia and North Africa; mostly growing in arid areas.

#### Notes

*Artemisia* sect. *Seriphidium* is circumscribed here similarly to the former *A.* subg. *Seriphidium* [10, 17], but excludes three annual species (i.e. *A. deserti*, *A. juncea* and *A. leucodes*), which are now placed in *A.* sect. *Junceae* (subg. *Absinthium*). A distinctive character of this section is the non-concurrent presence of florets and leaves—the leaves are mostly shed by flowering, a likely adaptation to arid habitats that complicates morphological observation. As the most drought-resistant lineage in *Artemisia*, species within sect. *Seriphidium* have diversified into distinct ecological niches; for example, *A. camelorum* and *A. pauciflora* are halophytes, whereas *A. santolina* is a psammophyte [45].

##### 6 *Artemisia* subg. *Tridentatae*

 (Rydb.) McArthur, Amer. J. Bot. 68: 590. 1981. ≡ *Artemisia* [unranked] *Tridentatae* Rydberg, N. Amer. Fl. 34: 282. 1916. – Type: *A. tridentata* Nutt. (Fig. 5).

#### Description

Subshrubs or shrubs, sometimes perennial herbs; 20–200 cm high. Root vertical. Stems numerous, sometimes few, erect. Leaves Type 1 (trilobed small leaves, at least the basal leaves trilobed). Synflorescence paniculate or racemose, rarely simple. Capitula of *Seriphidium* or *Artemisia* type, also rarely of *Absinthium* or *Dracunculus* type. Achenes without crown.

#### Species number and distribution

40 species; mainly distributed in western North America, with a few species in South America and Northeast Asia.

#### Notes

While the original *A.* subg. *Tridentatae* sensu McArthur et al. [46] is characterized by discoid capitula and a New World distribution, our phylogenetic analysis [1, 24] supported a broader circumscription. We here expand the subgenus to include *Sphaeromeria* and *Picrothamnus* from west North America as well as additional species from South America (e.g. *Artemisia sodiroi*), Beringia (e.g. *A. furcata*), and Northeast Asia (e.g. *A. lagocephala*). All newly included species are shrubs or subshrubs with 3-lobed or entire basal leaves.

##### 6.1 *Artemisia* sect. *Younghusbandianae*

 B.H.Jiao & T.G.Gao, **sect.** **nov.** – Type: *A. younghusbandii* J.R.Drumm. ex Pamp.

#### Description

Shrubs or subshrubs, 15–30 cm high. Root vertical, with thick woody stock and branches, much branched; densely gray tomentose. Leaves Type 3 (2-palmate medium leaves). Synflorescence paniclate. Capitula of *Absinthium* type. Disk floret corollas tubular; marginal floret corollas narrow tubular; style of disk florets bifid, apex truncate; style of marginal florets apex acute; anther apical appendages oblong; anther thecal base obtuse; anther collar oblong.

#### Species number and distribution

Only 1 species, *Artemisia younghusbandii*, endemic to Xizang, China.

#### Notes

*Artemisia younghusbandii* alone forms the most basal lineage within *A.* subg. *Tridentatae* [1]. Although originally placed in *A.* subg. *Absinthium* sensu Ling et al. [3] based on its pubescent receptacle, our phylogenetic analyses firmly nest it within *A.* subg. *Tridentatae*, showing close relationship to Beringian and New World species such as *A. lagocephala*. Morphologically, *A. younghusbandii* shares with other members of the subgenus—including *A. lagocephala*—the presence of 3-lobed uppermost leaves and a pubescent receptacle, characters consistent with the circumscription of *A.* subg. *Tridentatae* sensu McArthur et al. [46]. Further morphological study and expanded sampling are needed to fully clarify relationships and character evolution within this section.

##### 6.2 *Artemisia* sect. *Lagocephalae*

 (Kitam.) B.H.Jiao & T.G.Gao, **stat.** **nov.** ≡ *Artemisia *ser. *Lagocephalae* Kitam. in Act. Phytotax. Geobot. 8: 64. 1939. ≡ *Artemisia *ser. *Crossostephioides* Poljakov, Fl. USSR 26: 504. 1961. – Type: *A. lagocephala* (Fisch. ex Besser) DC. = *Artemisia *ser. *rutifoliae* Poljakov, Fl. USSR 26: 506. 1961. – Type: *A. rutifolia* Willd. ex Spreng.

#### Description

Perennials or subshrubs, 40–60 cm high. Root vertical, with thick woody stock and branches, much branched; densely gray tomentose. Leaves Type 1 (trilobed small leaves). Synflorescence racemose or paniculate. Capitula of *Absinthium* type. Disk floret corollas campanulate tubular; marginal floret corollas narrow tubular; style of disk florets bifid, apex truncate; style of marginal florets apex acute; anther apical appendages oblong; anther thecal base tailed; anther collar oblong or balusterform.

#### Species number and distribution

3 species; distributed in Arctic, Siberia, and Far East regions of Asia.

#### Notes

*Artemisia* sect. *Lagocephalae* is here established by raising *A.* ser. *Lagocephalae* sensu Kitamura to sectional rank, with the addition of *A. rutifolia* and *A. kruhsiana*. It is characterized by the *Absinthium*-type capitulum (pubescent receptacle), a character shared with most species of *A.* subg. *Absinthium*. However, its members share leaf type 1 with other species of *A.* subg. *Tridentatae*, supporting its placement within this subgenus.

##### 6.3 *Artemisia* sect. *Tridentatae*

 L.M.Shultz, Syst. Bot. Monogr. 89: 33. 2009. – Type: *A. tridentata* Nutt. = *Artemisia* sect. *Nebulosae* L. M. Shultz, Syst. Bot. Monogr. 89: 97. 2009. – Type: *Artemisia californica* Lessing = *Artemisia* sect. *Filifoliae* (Rydb.) Sòn.Garcia, Garnatje, McArthur, Pellicer, S.C.Sand. & Vallès-Xirau, W. N. Amer. Naturalist 71(2): 159. 2011. = *Artemisia* [unranked] *Filifoliae* Rydberg, N. Amer. fl. 34: 257. 1916.—Type: *Artemisia* *filifolia* Torrey. = *Artemisia* [unranked] *Franserioides* Rydberg, N. Amer. fl. 34: 265. 1916. – Type: *A. franserioides* Greene = *Artemisia* [unranked] *Pygmaea* Rydberg, N. Amer. fl. 34: 285. 1916. – Type: *A*. *pygmaea* A.Gray =* Artemisia* [unranked]* Rigidae* Rydberg, N. Amer. fl. 34: 284. 1916. – Type: *A. rigida* (Nutt.) A.Gray =* Artemisia* [unranked]* Bigelovianae* Rydberg, N. Amer. fl. 34: 281. 1916. – Type: *A. bigelovii* A.Gray

#### Description

Shrubs or subshrubs; 20–200 cm high. Root vertical, with thick woody stock and branches, much branched. Leaves Type 1 (trilobed small leaves, or entire). Synflorescence racemose or paniculate, rarely with only one capitulum. Capitula of *Artemisia* or *Tridentatae* type, rarely *Dracunculus* type (*A. filifolia*). Disk floret corollas campanulate tubular, cup shaped tubular or tubular; marginal floret corollas narrow tubular; style of disk florets bifid, apex truncate or undivided; style of marginal florets apex acute or retuse; anther apical appendages oblong, acute or attenuate; anther thecal base obtuse, sagittate or tailed; anther collar oblong.

#### Species number and distribution

36 species; western North America, the Arctic and temperate regions of South America.

#### Notes

*Artemisia* sect. *Tridentatae* constitutes the core of *A.* subg. *Tridentatae*. Here we expand its circumscription to include 27 species previously placed in *A.* subg. *Artemisia* or unplaced. While capitulum and synflorescence types vary within the section (see Jiao et al. [1], Fig. 5), all examined species possess trilobed leaves. In *A. porteri*, such leaves are basal and mostly missing from specimens [47], likely accounting for reports of their absence. Further morphological study of the newly added species is warranted.

##### 7 *Artemisia* subg. *Absinthium*

 (Miller) Lessing, Linnaea 6(2): 217. 1831. ≡ *Absinthium* Miller, Gard. Dict. Abr. ed. 4, vol. 1. 1754. – Type: *A. absinthium* L. (Fig. 5).

#### Description

Annuals, biennials, perennials, or rarely shrubs; 20–150 cm high. Stems erect, prostrate, or rosette. Leaves show significant variations, including Types 1, 2, 3, 4, 9, 10. Synflorescence paniculate or racemose. Capitula of *Absinthium* type, rarely *Seriphidium* type (sect. *Junceae*) or *Artemisia* type (sect. *Blepharolepides* and some other species). Achenes without crown and ribs.

#### Species number and distribution

64 species; mainly in the temperate or cold temperate regions of Eurasia, a few in North America.

#### Notes

The traditional delineation of *A.* subg. *Absinthium* relied on the *Absinthium* type capitula (with pubescent receptacles), yet the taxonomic value of this character has long been debated [48]. Our reconstruction of capitulum evolution confirms that this character has originated multiple times independently within *Artemisia* [1, 24]. Accordingly, we have revised the circumscription of the subgenus by excluding short annual halophytes (*A.* sect. *Anethifoliae*) plus shrubby species with 3-lobed or pinnatisect leaves (*A.* sect. *Younghusbandianae* and sect. *Lagocephalae*), and adding annual species with *Seriphidium* type capitula (sect. *Junceae*) plus an annual species with *Artemisia* type capitula (sect. *Blepharolepides*). The recircumscribed *A.* subg. *Absinthium* is best defined by a combination of the *Absinthium*-type capitulum, globose capitulum shape, and leaves that are medium-sized and 2-palmate. Despite its relatively small number of species, this subgenus exhibits the widest ecological amplitude within *Artemisia*. It ranges from the common temperate weed *A. sieversiana* and the dominant Eurasian grassland species *A. frigida*, to the drought-tolerant desert dweller *A. xerophytica*, the alpine cushion-plant *A. minor*, and the island endemic *A. thuscula*. This remarkable ecological diversity makes *A.* subg. *Absinthium* an outstanding system for studying adaptive evolution.

##### 7.1 *Artemisia* sect. *Blepharolepides*

 (Y.R.Ling) B.H.Jiao & T.G.Gao, **stat.** **nov.** ≡ *Artemisia* ser. *Blepharolepides* Y.R.Ling, Bull. Bot. Res. 8(4): 54. 1988. – Type: *A. blepharolepis* Bge.

#### Description

Annuals or biennials; 20–60 cm high. Root vertical. Stems few, many branches, erect. Leaves Type 4 (small, 2-pinnatisect, multiple-lobed). Synflorescence paniclate. Capitula of *Artemisia* type; corolla dark yellow. Disk floret corollas cup shaped tubular; marginal floret corollas narrow tubular; style of disk florets bifid, apex truncate; style of marginal florets apex retuse; anther apical appendages acute; anther thecal base sagittate; anther collar balusterform.

#### Species number and distribution

Only 1 species; Northern China and Mongolia.

#### Notes

*Artemisia blepharolepis* was previously placed in *A.* subg. *Dracunculus* as its disk florets were regarded as functionally staminate [3]. Our examination of herbarium specimens and field observations, however, confirms that its disk florets are bisexual and fertile. Furthermore, nuclear phylogenetic data further place it not in *A.* subg. *Dracunculus*, but as a basal lineage within *A.* subg. *Absinthium* [1]. Unlike the typical *Absinthium*-type, its receptacles are glabrous—a character consistent with the finding that receptacle pubescence has evolved multiple times independently in *Artemisia*. The species is readily identified by its narrow synflorescences, finely dissected leaves, and fast-growing annual habit, typically completing germination, flowering, and fruiting rapidly after summer rains.

##### 7.2 *Artemisia* sect. *Sieversianae*

 (Kitam.) B.H.Jiao & T.G.Gao, **stat.** **nov.** ≡ *Artemisia* subsect. *Sieversianae* Kitam., Act. Phytotax. Geobot. 8: 66. 1939. ≡ *Artemisia* ser. *Sieversianae* (Kitam.) Y.R.Ling, Bull. Bot. Res. 8(4): 4. 1988. – Type: *A. sieversiana* Ehrh. ex Willd.

#### Description

Annuals or biennials. Root vertical. Stems single, erect, more than 100 cm high; or few, prostrate, 15–30 cm high. Leaves Type 3 (small 2-palmate medium leaves), and Type 10 (large, pinnatisect broad-lobed). Synflorescence paniclate. Capitula of *Absinthium* or rarely *Artemisia* type (*A. shangnanensis*), oblate or globose, 5–10 mm in diam. Disk floret corollas campanulate tubular; marginal floret corollas conical; style of disk florets bifid, apex truncate; style of marginal florets apex retuse; anther apical appendages acute, attenuate or oblong; anther thecal base obtuse or sagittate; anther collar balusterform.

#### Species number and distribution

7 species; *Artemisia sieversiana* widely distributed in temperate Asia, others in Central Asia and Northern Asia.

#### Notes

*Artemisia* sect. *Sieversianae* is characterized by large, globose capitula. Morphologically, *A. succulentoides* fits well within this section. However, it is known only from type collections, and we were unable to relocate it in its type locality (Xizang) despite repeated attempts. Its taxonomic placement therefore requires further study.

##### 7.3 *Artemisia* sect. *Absinthium*

 (Mill.) DC., Fl. Franc. [de Candolle & Lamarck], ed. 3. 4(1): 189. 1805. ≡ *Absinthium* Miller, Gard. Dict. Abr. ed. 4, vol. 1. 1754. – Type: *A. absinthium* L.

#### Description

Perennials, shrubs or subshrubs. Root vertical. Stems single or few, erect, more than 100 cm high. Leaves Type 9 (medium to big, pinnatisect, lobe ovate). Synflorescence paniclate. Capitula of *Absinthium* type, oblate or globose. Disk floret corollas campanulate tubular; marginal floret corollas conical; style of disk florets bifid, apex truncate; style of marginal florets apex acute; anther apical appendages acute; anther thecal base Sagittate or obtuse; anther collar balusterform.

#### Species number and distribution

5 species; *Artemisia absinthium* widely distributed in Central Asia, Europe, and North Africa, *A. arborescens* in the Mediterranean area, the other three species endemic to Atlantic Macaronesian islands, including *A. thuscula* from the Canary Islands, *A. argentea* from the Madeira Islands and *A. gorgonum* from Cape Verde.

#### Notes

*Artemisia* sect. *Absinthium* includes only *A. absinthium* and its close relatives. It is sister to sect. *Sieversianae*, with both sections sharing large, globose capitula. They differ, however, in life form and distribution: sect. *Absinthium* is shrubby and occurs in western Eurasia and the adjacent Macaronesian islands off Africa, while sect. *Sieversianae* consists of annual herbs endemic to eastern Eurasia.

##### 7.4 *Artemisia* sect. *Junceae*

 Poljakov ex Filatova, Novosti Sist. Vyssh. Rast. 23: 219. 1986. – Type: *A.* *juncea* Kar. & Kir.

#### Description

Annuals, or perennials. Stem numerous, erect, 30–60 cm high. Leaves Type 1 (trilobed small leaves, or entire). Synflorescence paniclate. Capitula of *Seriphidium* type, oblong. Disk floret corollas cup shaped tubular; marginal floret corollas absent; style of disk florets bifid, apex truncate; style of marginal florets absent; anther apical appendages oblong; anther thecal base obtuse; anther collar oblong.

#### Species number and distribution

4 species; Central Asia

#### Notes

*Artemisia* sect. *Junceae* is characterized by having *Seriphidium* type capitula and entire or 3-lobed leaves. Although previously placed in *A.* subg. *Seriphidium* sensu Poljakov [17], it can be distinguished from the core *Seriphidium* lineage (here treated as sect. *Seriphidium*) by leaf morphology: species in sect. *Junceae* possess Type 1 leaves, whereas those in sect. *Seriphidium* have Type 2 leaves.

##### 7.5 *Artemisia* sect. *Frigidae*

(Rydb.) B.H.Jiao & T.G.Gao, **stat.** **nov.** ≡ *Artemisia *[unranked] *Frigidae* Rydb. N. Amer. Fl. 34 (3): 258. 1916. – Type: *A. frigida* Willd. = *Artemisia* [unranked] *Glomeratae *Rydberg, N. Amer. fl. 34: 260. 1916. – Type: *A. glomerata* Ledeb. = *Artemisia *ser.* Rupestres* Poljakov, Fl. URSS 26: 509. 1961. – Type: *A. rupestris *L.= *Artemisia *ser.* Obtusilobae* Poljakov, Fl. URSS 26: 510. 1961. – Type: *A. obtusiloba* Ledeb.

#### Description

Perennials, rarely annuals or shrubs. Stem numerous, erect, 10–40 cm high, tomentose or puberulent. Leaves Type 3 (2-palmate small to medium leaves), rarely Type 2 (small, pinnatisect multiple-lobed). Synflorescence paniculate or racemose, rarely single. Capitula of *Absinthium* or rarely *Artemisia* type; ovoid or oblate. Disk floret corollas campanulate tubular; marginal floret corollas narrow tubular; style of disk florets bifid, apex truncate; style of marginal florets apex acute; anther apical appendages oblong or acute; anther thecal base sagittate, tailed or obtuse; anther collar oblong or balusterform.

#### Species number and distribution

48 species; North America, South America and Eurasia

#### Notes

*Artemisia* sect. *Frigidae* forms the core of *A.* subg. *Absinthium*. It is characterized by ovoid capitula, 2-palmate small to medium leaves, and a densely hairy indumentum. Many species in this section exhibit characters highly adapted to alpine habitats: short stature, dense hair cover, and short, narrow synflorescences. As reported by Mas de Xaxars et al. [49], the chromosome base number in this alpine members this section has shifted from the ancestral *x* = 9 to *x* = 8, with most species being diploid and only few polyploid. This suggests that a change in base number, rather than polyploidization, may have been a key factor in the adaptation of sect. *Frigidae* to harsh alpine environments.

##### 8 *Artemisia* subg. *Artemisia*.

 – Type: *A. vulgaris* L. (Fig. 6).

#### Description

Perennials. Stems erect; 20–150 cm high. Leaves large, Type 10 (medium to large, pinnatisect broad-lobed), rarely Type 7 (entire medium leaves, but not toothed), Type 8 (large, 5-lobed to pinnatipartite); if not entire, the width of lobes > 2 mm. Synflorescence paniculate or racemose. Capitula of *Artemisia* type. Achenes without crown and ribs.

#### Species number and distribution

111 species; mainly in East Asia, a few extending to Europe and the New World.

#### Notes

*Artemisia* subg. *Artemisia* is the only major lineage in the genus that consistently occupies moist habitats. Traditionally defined by glabrous receptacles, disciform capitula, and bisexual, fertile disk florets—a combination termed the “*Artemisia* type”, representing the ancestral state for the genus [1]—this subgenus historically included all species with this capitulum type, often without phylogenetic support [3, 17, 50]. Here, based on combined morphological and phylogenetic evidence, we redefine a more narrowly circumscribed *A.* subg. *Artemisia*, having transferred multiple species to *A.* subg. *Dracunculus*, *A.* subg. *Pectinatae*, *A.* subg. *Ponticae*, *A.* subg. *Seriphidium*, and *A.* subg. *Tridentatae*. Notably, phylogenetic signals from nuclear and chloroplast genomes conflict: chloroplast data resolve *A.* subg. *Artemisia* as non-monophyletic, splitting into two clades (Jiao et al. [1]) that correspond respectively to *A.* sect. *Selengenses* and *A.* sect. *Artemisia* (Jiao et al. [1]).

##### 8.1 *Artemisia* sect. *Selengenses*

 (Pamp.) B.H.Jiao & T.G.Gao, **stat.** **nov.** ≡ *Artemisia* [infragen.unranked] *Selengenses* Pamp., Nuov. Giorn. Bot. Ital. n.s. 36: 500. 1930. – Type: *A. selengensis* Turcz. ex Besser.

#### Description

Perennial herbs, sometimes subshrubs; 70–120 cm high; rootstock horizontally creeping to obliquely rising. Leaves medium to large, Type 7 (entire leaves, but not toothed), or Type 8 (5-lobed to pinnatipartite). Synflorescence paniculate, rarely racemose. Capitula of *Artemisia* type, oblong, ovoid, or ovoid-campanulate; 2–5 mm in diam. Disk floret corollas cup shaped tubular; marginal floret corollas filiform; style of disk florets bifid, apex truncate; style of marginal florets apex acute; anther apical appendages attenuate; anther thecal base tailed or sagittate; anther collar balusterform.

#### Species number and distribution

14 species; Northeast Asia and North America.

#### Notes

*Artemisia* sect. *Selengenses* includes some East Asian species such as *A. anomala* and *A. viridissima*, and some North American species such as *A. ludoviciana* (= *A. vulgaris* sensu Keck) complex [48].

##### 8.2 *Artemisia* sect. *Artemisia*

 ≡ *Artemisia* [unranked] *Vulgares* Rydberg, N. Amer. fl. 34: 265. 1916. – Type: *A.* *vulgaris *L. = *Artemisia *sect. *Albibractea* Y. R. Ling, Act. Phytotax. Sin. 18(4): 506. 1980. – Type: *A. lactiflora *Wall. ex DC.* = Artemisia* sect. *Viscidipubes *Y. R. Ling, Act. Phytotax. Sin. 18(4): 506. 1980. – Type: *A. viscida *Pamp. *= Artemisia* sect. *Stellerianum* (Rydb.) Poljakov, Fl. URSS 26: 433. 1961. = *Artemisia* [unranked] *Stellerianae* Rydberg, N. Amer. fl. 34: 277. 1916. – Type: *A.* *stellerianae *Besser = *Artemisia* ser. *Umbrosae* (Pamp.) Y. R. Ling, Bull. Bot. Res. 8(4): 21. 1988. = *Artemisia* [infragen.unranked] *Umbrosae* Pamp., Nuov. Giorn. Bot. Ital. n.s. 36: 506. 1930. – Type: *A. umbrosa* (Besser) Turcz. ex Verl. = *Artemisia* ser. *Codonocephalae* (Pamp.) Y. R. Ling, Bull. Bot. Res. 8(4): 22. 1988. = *Artemisia* [infragen.unranked] *Codonocephalae* Pamp., Nuov. Giorn. Bot. Ital. n.s. 36: 507. 1930. – Type: *A. codonocephala *Diels = *Artemisia* ser. *Microcephalae* (Kitam.) Y. R. Ling, Bull. Bot. Res. 8(4): 24. 1988*. *= *Artemisia* subser. *Microcephalae* Kitam., Mem. Coll. Sci. Kyoto Univ. ser. B. 15(3): 410. 1940. – Type: *A. feddei* H.Lév. & Vaniot = *Artemisia* [infragen.unranked] *Mongolicae* Pamp., Nuov. Giorn. Bot. Ital. n.s. 36: 499. 1930. – Type: *A. mongolica *(Fisch. ex Besser) Nakai = *Artemisia* ser. *Igniariae* (Pamp.) Y. R. Ling, Bull. Bot. Res. 8(4): 30. 1988. = *Artemisia* [infragen.unranked] *Igniariae* Pamp., Nuov. Giorn. Bot. Ital. n.s. 36: 505. 1930. – Type: *A. igniaria* Maxim. = *Artemisia* ser. *Monophyllae* (Kitam.) Y. R. Ling, Bull. Bot. Res. 8(4): 32. 1988*. *= *Artemisia* subsect. *Monophyllae* Kitam., Mem. Coll. Sci. Kyoto Univ. ser. B. 15(3): 413. 1940. – Type: *A. monophylla* Kitam. = *Artemisia* ser. *Moorcroftianae* (Pamp.) Y. R. Ling, Bull. Bot. Res. 8(4): 32. 1988. ≡ *Artemisia* [infragen.unranked] *Moorcroftianae* Pamp., Nuov. Giorn. Bot. Ital. n.s. 36: 502. 1930. – Type: *A. moorcroftiana* Wall. ex DC. = *Artemisia* ser. *Serpens* (Kitam.) Y. R. Ling, Bull. Bot. Res. 8(4): 34. 1988*. *= *Artemisia* subsect. *Serpens* Kitam., Act. Phytotax. Geobot. 8: 63. 1939. – Type: *A. somae *Hayata = *Artemisia* ser. *Fulgentes* (Pamp.) Y. R. Ling, Bull. Bot. Res. 8(4): 36. 1988. = *Artemisia* [infragen.unranked] *Fulgentes* Pamp. Nuov. Giorn. Bot. Ital. n.s. 36: 503. 1930. – Type: *A. fulgens *Pamp. *= Artemisia* ser. *Silvaticae* Poljakov, Fl. URSS 26: 445. 1961. – Type: *A. sylvatica *Maxim. = *Artemisia* ser. *Pleiocephalae* (Pamp.) Y. R. Ling, Bull. Bot. Res. 8(4): 38. 1988. = *Artemisia* [infragen.unranked] *Pleiocephae* Pamp., Nuov. Giorn. Bot. Ital. n.s. 36: 506. 1930. – Type: *A. pleiocephala* Pamp.

#### Description

Perennial herbs, sometimes subshrubs; 30–150 cm high; rootstock horizontally creeping to obliquely rising. Leaves large, Type 10 (pinnatisect broad-lobed), rarely Type 7 (entire leaves, but not toothed), Type 8 (5-lobed to pinnatipartite). Synflorescence paniculate, rarely racemose; capitula of *Artemisia* type; oblong, ovoid, or ovoid-campanulate; 2–5 mm in diam. Disk floret corollas cup shaped tubular; marginal floret corollas filiform; style of disk florets bifid, apex truncate; style of marginal florets apex acute; anther apical appendages attenuate or acute; anther thecal base tailed or sagittate; anther collar balusterform, rarely oblong.

#### Species number and distribution

97 species; widely distributed in the temperate regions of Eurasia, a few extending into the tropical regions of Southeast Asia.

#### Notes

The recircumscribed *A.* sect. *Artemisia* forms the core of *A.* subg. *Artemisia*. It approximately corresponds to the combination of sect *Artemisia*, sect. *Albibractea*, and sect. *Viscidipubes* sensu Ling et al. [3], which were historically distinguished by white phyllary (sect. *Albibractea*) and glandular pubescence (sect. *Viscidipubes*). However, phylogenetic analyses do not support the monophyly of these previously recognized sections (Fig. 6), indicating that phyllary color and indumentum type are not reliable taxonomic characters at the sectional level within this subgenus.

Natural hybridization among species in this section—for example, between *A. gilvescens* and *A. indica* var. *maximowiczii* [51]—has been reported. Additionally, all species of *A.* sect. *Artemisia* possess well-developed rhizomes and the ability to reproduce asexually, characters that may facilitate the rapid fixation and spread of genetic variants. Although phylogenetic analysis indicates that this section can be divided into two subgroups (Fig. 6), no consistent morphological differences currently support such a split.

#### Unplaced species of *Artemisia*

*Artemisia avarica* Minatul., *A. dipsacea* Krasch., *A. galinae* Ikonn. These three species remain taxonomically unplaced due to the absence of reliable morphological or molecular evidence, which stems from vague protologues and the lack of accessible specimens.

## Conclusions

This study establishes the first comprehensive sectional taxonomy for the genus *Artemisia* on a global scale. By systematically delineating twenty-four manageable sections from a big and complex genus of 505 species, this framework provides a robust baseline for future research into this medically, ecologically, and economically important group. Most sections are confined to specific countries or regions, making them practical units for local taxonomists to revise and study within a feasible timeframe. More broadly, the study presents a methodological case for addressing taxonomic complexity in other big plant genera.The detailed sectional hypotheses proposed here will further facilitate applied studies, such as the discovery of potential new medicinal compounds beyond artemisinin, the identification of suitable taxa for desertification control, and the characterization of pollen-derived proteins for allergen-specific immunotherapy.

Moreover, the new taxonomy reveals notable adaptive patterns among the major lineages of *Artemisia*. Different subgenera exhibit notable niche differentiation across geographic regions: for instance, *A.* subg. *Pacifica* is restricted to coastal Southeast Asia and the subalpine zones of Hawaii, *A.* subg. *Seriphidium* is centered in the arid regions of Central Asia, while *A.* subg. *Absinthium* demonstrates broad ecological adaptability, occurring from temperate grasslands to alpine meadows. These biogeographic and ecological insights lay the groundwork for future research into the correlation between the evolution of key morphological characters—such as leaf size, plant height, and capitulum type—and environmental factors like drought, low temperature, and salinity. Such work will establish *Artemisia* as an informative model for understanding the adaptation and evolution of plants.

Several aspects of the proposed taxonomy highlight priorities for future research. First, the placement of three species remains unresolved. Second, species-rich and taxonomically complex sections—notably *A.* sect. *Latilobus* (Fig. 3), *A.* sect. *Artemisia* (Fig. 6), and *A.* sect. *Seriphidium* (Fig. 4)—require denser sampling and integrated phylogenomic-morphological analyses to clarify species boundaries. Finally, as our phylogeny employed a single representative per species, it does not address species delimitation in *Artemisia*. Consequently, the species treatment and the key presented here constitute a working framework, reflecting the dynamic and iterative process of taxonomic research. We plan to reassess this framework every 2–3 years and will update it once substantial new evidence has emerged from ongoing and future studies.

## Supplementary Information

Supplementary Material 1. List of taxa included in the phylogenetic analysis, with associated herbarium voucher details and GenBank accession numbers.

Supplementary Material 2. Taxonomic inventory of *Artemisia* species, detailing their subgeneric and sectional placements.

## Data Availability

The data supporting this article have been included in the manuscript and supplementary information. Datasets analysed in this study are available in figshare data repository (10.6084/m9.figshare.28164335).

## Abbreviations

AIC: Akaike information criterion
BS: Bootstrap Support
CoL: Catalog of Life
DNA: Deoxyribonucleic Acid
ETS: External Transcribed Spacer
FAA: Formalin—Acetic—Alcohol
GCC: Global Compositae Checklist
ICN: International Code of Nomenclature for algae, fungi, and plants
ITS: Internal Transcribed Spacer
ML: Maximum likelihood
nom. nov.: Nomen novum
POWO: Plants of the World Online
sect. nov.: Sectio nova
SNP: Single nucleotide polymorphisms
stat. nov.: Status novus
WFO: World Flora Online

## References

[1] Jiao B, Wei M, Niu G, Chen X, Liu Y, Huang G, et al. Global phylogeny and taxonomy of *Artemisia*. Nat Commun. 2025;16(1):8648. 41062471 10.1038/s41467-025-64039-0 PMC12508166

[2] Moonlight PW, Baldaszti L, Cardoso D, Elliott A, Sarkinen T, Knapp S. Twenty years of big plant genera. Proc Biol Sci. 2024;291(2023):20240702. 38808446 10.1098/rspb.2024.0702 PMC11285793

[3] Ling Y, Humphries CJ, Gilbert MG. *Artemisia* L. In: Wu ZY, Raven P, Hong DY, editors. Flora of China, vol. 20. Beijing, St. Louis: Science Press, Missouri Botanical Garden Press; 2011. p. 1151–259.

[4] Shultz LM, Flora of North America Editorial Committee. *Artemisia*. In: Flora of North America. New York: Oxford University Press; 2006. p. 503–34.

[5] Vallés J, Garcia S, Hidalgo O, Martín J, Pellicer J, Sanz M, Garnatje T: Biology, Genome Evolution, Biotechnological Issues and Research Including Applied Perspectives in *Artemisia* (Asteraceae). In: *Advances in Botanical Research.* Edited by Kader JC, Delseny M. 2011;60:349–419.

[6] Torrell M, Garcia-Jacas N, Susanna A, Vallés J. Phylogeny in *Artemisia* (Asteraceae, Anthemideae) inferred from nuclear ribosomal DNA (ITS) sequences. Taxon. 1999;48(4):721–36.

[7] Vallés J, Torrell M, Garnatje T, Garcia-Jacas N, Vilatersana R, Susanna A. The genus *Artemisia* and its allies: phylogeny of the subtribe Artemisiinae (Asteraceae, Anthemideae) based on nucleotide sequences of nuclear ribosomal DNA internal transcribed spacers (ITS). Plant Biol. 2003;5(3):274–84.

[8] Pellicer J, Garnatje T, Molero J, Pustahija F, Siljak-Yakovlev S, Valles J. Origin and evolution of the South American endemic *Artemisia* species (Asteraceae): evidence from molecular phylogeny, ribosomal DNA and genome size data. Aust J Bot. 2010;58(7):605–16.

[9] Pellicer J, Vallés J, Korobkov AA, Garnatje T. Phylogenetic relationships of *Artemisia* subg. *Dracunculus* (Asteraceae) based on ribosomal and chloroplast DNA sequences. Taxon. 2011;60(3):691–704.

[10] Malik S, Vitales D, Hayat MQ, Korobkov AA, Garnatje T, Vallès J. Phylogeny and biogeography of *Artemisia* subgenus *Seriphidium* (Asteraceae: Anthemideae). Taxon. 2017;66(4):934–52.

[11] Linnaeus C. *Artemisia*. In: Species plantarum, vol. 2. Stockholm: Laurentii Salvii; 1753. p. 845–50.

[12] Martin J, Torrell M, Vallès J. Palynological features as a systematic marker in *Artemisia* L. and related genera (Asteraceae, Anthemideae). Plant Biol. 2001;3(4):372–8.

[13] Bremer K, Humphries CJ. Generic monograph of the Asteraceae - Anthemideae. Bulletin of the Natural History Museum London (Botany). 1993;23(2):71–177.

[14] Linnaeus C: *Artemisia*. In: *Genera plantarum.* Stockholm: Laurentii Salvii; 1754: 850.

[15] Linnaeus C. *Artemisia*. In: Systema naturae, vol. 2. Stockholm: Direct; 1767. p. 541–3.

[16] Ghafoor A. *Artemisiella*, a new genus of Compositae based on *Artemisia stracheyi* Hook.f. & Thorns, ex Clarke. Candollea. 1992;47:635–43.

[17] Poljakov PP. *Artemisia* L. In: Shishkin BKBE, editor. Flora of the USSR, vol. 26. Leningrad: Nauka; 1961. p. 425–631.

[18] Poljakov PP. Materials for the systematics of the genus *Artemisia*. Trudy Inst Bot (Alma - Ata). 1961;11:134–78.

[19] Watson LE, Bates PL, Evans TM, Unwin MM, Estes JR. Molecular phylogeny of Subtribe Artemisiinae (Asteraceae), including *Artemisia* and its allied and segregate genera. BMC Evol Biol. 2002. 10.1186/1471-2148-2-17. 12350234 10.1186/1471-2148-2-17 PMC130036

[20] Sanz M, Vilatersana R, Hidalgo O, Garcia-Jacas N, Susanna A, Schneeweiss GM, et al. Molecular phylogeny and evolution of floral characters of *Artemisia* and allies (Anthemideae, Asteraceae): evidence from nrDNA ETS and ITS sequences. Taxon. 2008;57(1):66–78.

[21] Garcia S, McArthur ED, Pellicer J, Sanderson SC, Vallès J, Garnatje T. A molecular phylogenetic approach to western North America endemic *Artemisia* and allies (Asteraceae): untangling the sagebrushes. Am J Bot. 2011;98(4):638–53. 21613164 10.3732/ajb.1000386

[22] Garcia S, Garnatje T, McArthur ED, Pellicer J, Sanderson SC, Vallès J. Taxonomic and nomenclatural rearrangements in *Artemisia* subgen. *Tridentatae*, including a redefinition of *Sphaeromeria* (Asteraceae, Anthemideae). West N Am Nat. 2011;71(2):158–63.

[23] Hobbs CR, Baldwin BG. Asian origin and upslope migration of Hawaiian *Artemisia* (Compositae-Anthemideae). J Biogeogr. 2013;40(3):442–54.

[24] Jiao B, Chen C, Wei M, Niu G, Zheng J, Zhang G, et al. Phylogenomics and morphological evolution of the mega-diverse genus *Artemisia* (Asteraceae: Anthemideae): implications for its circumscription and infrageneric taxonomy. Ann Bot. 2023;131(5):867–83. 36976653 10.1093/aob/mcad051 PMC10184459

[25] Wei M, Jiao B-H, Niu G-H, Chen X-Y, Leostrin A, Seregin PA, et al. Merging *Ajaniopsis* (Asteraceae), an endangered genus endemic to the Tibet Plateau, into *Artemisia*: implications for systematics and conservation. Taxon. 2024;74:417–34.

[26] Munoz-Rodriguez P, Wood JRI, Wells T, Carruthers T, Sumadijaya A, Scotland RW. The challenges of classifying big genera such as *Ipomoea*. Taxon. 2023;72(6):1201–15. 40687846 10.1002/tax.12887 PMC7617906

[27] Criado-Ruiz D, Vallès J, Bayer RJ, Palazzesi L, Pellicer J, Lorenzo IP, et al. A phylogenomic approach to disentangling the evolution of the large and diverse daisy tribe Anthemideae (Asteraceae). J Syst Evol. 2024;63:282–306.

[28] Portik DM, Streicher JW, Blackburn DC, Moen DS, Hutter CR, Wiens JJ. Redefining possible: combining phylogenomic and supersparse data in frogs. Mol Biol Evol. 2023;40(5):msad109. 37140129 10.1093/molbev/msad109 PMC10202597

[29] Minh BQ, Schmidt HA, Chernomor O, Schrempf D, Woodhams MD, von Haeseler A, et al. IQ-TREE 2: new models and efficient methods for phylogenetic inference in the genomic era. Mol Phylogenet Evol. 2020;37:1530–4. 10.1093/molbev/msaa015 PMC7182206 32011700

[30] Kalyaanamoorthy S, Bui Quang M, Wong TKF, von Haeseler A, Jermiin LS. ModelFinder: fast model selection for accurate phylogenetic estimates. Nat Methods. 2017;14(6):587–9. 28481363 10.1038/nmeth.4285 PMC5453245

[31] Roque N, Keil DJ, Susanna A. Illustrated glossary of Compositae. In: Funk V, Stuessy T, Bayer R, editors. Systematics, evolution and biogeography of the Compositae. Vienna: International Association Plant Taxonomy; 2009. p. 781–806.

[32] Grossi MA, Viera Barreto JN, Plos A, Rodríguez-Cravero JF, Forte NB, Gutiérrez DG, et al. Providing tools for the reassessment of *Eupatorieae* (Asteraceae): comparative and statistical analysis of reproductive characters in South American taxa. Perspect Plant Ecol Evol Syst. 2020;46:125566.

[33] Blomberg SP, Garland T, Ives AR. Testing for phylogenetic signal in comparative data: behavioral traits are more labile. Evolution. 2003. 10.1554/0014-3820(2003)057[0717:TFPSIC]2.0.CO;2. 12778543 10.1111/j.0014-3820.2003.tb00285.x

[34] Pagel M. Inferring the historical patterns of biological evolution. Nature. 1999;401:877–84. 10553904 10.1038/44766

[35] R-Core-Team. R: A language and environment for statistical computing. Vienna, Austria: R Foundation for Statistical Computing; 2019.

[36] Muñoz-Rodríguez P, Wood JRI, Wells T, Carruthers T, Sumadijaya A, Scotland RW. The challenges of classifying big genera such as *Ipomoea*. Taxon. 2023;72(6):1201–15. 40687846 10.1002/tax.12887 PMC7617906

[37] Shultz LM. Monograph of *Artemisia* Subgenus *Tridentatae* (Asteraceae-Anthemideae). Syst Bot Monogr. 2009;89(Oct. 19):1–131.

[38] Turland NJ, Wiersem JH, Barri FR, Gandhi KG, Gravendyck J, Greuter W, et al., editors. International Code of Nomenclature for algae, fungi, and plants (Madrid Code) adopted by the Twentieth International Botanical Congress Madrid, Spain, July 2024. Chicago: University of Chicago Press; 2025.

[39] Pellicer J, Garcia S, Canela MA, Garnatje T, Korobkov AA, Twibell JD, et al. Genome size dynamics in *Artemisia* L. (Asteraceae): following the track of polyploidy. Plant Biol. 2010;12(5):820–30. 20701707 10.1111/j.1438-8677.2009.00268.x

[40] Garcia S, Sanz M, Garnatje T, Kreitschitz A, McArthur ED, Vallès J. Variation of DNA amount in 47 populations of the subtribe Artemisiinae and related taxa (Asteraceae, Anthemideae): karyological, ecological, and systematic implications. Genome. 2004;47(6):1004–14. 15644958 10.1139/g04-061

[41] Ling YR. The Old World *Artemisia* L. (Compositae). Bull Bot Res. 1992;12(1):1–108.

[42] Mahoney KJ, Kegode GO. Biennial wormwood (*Artemisia biennis*) biomass allocation and seed production. Weed Sci. 2017;52(2):246–54.

[43] Ling YR. The Chinese *Artemisia* L.——the classification, distribution and application of *Artemisia* L. in China. Bull Bot Res. 1988;8(4):1–61.

[44] Tkach NV, Hoffmann MH, Roser M, Korobkov AA, von Hagen KB. Parallel evolutionary patterns in multiple lineages of arctic *Artemisia* L. (Asteraceae). Evolution. 2008;62(1):184–98. 17976192 10.1111/j.1558-5646.2007.00270.x

[45] Safronova IN: Species of *Artemisia* subgenus *Seriphidium* in the West Turan and their ecology. In: *Compositae: Biology & Utilization Proceedings of the International Compositae Conference, Kew, 1994.* Edited by Caligari PDS, Hind DJN, vol. 2: Royal Botanic Gardens, Kew; 1996: 105–110.

[46] McArthur E, Pope C, Freeman D. Chromosomal studies of subgenus *Tridentatae* of *Artemisia*: evidence for autopolyploidy. Am J Bot. 1981;68:589–605.

[47] Cronquist A. A new *Artemisia* from Wyoming. Madroño. 1951;11:145–6.

[48] Riggins CW, Seigler DS. The genus *Artemisia* (Asteraceae: Anthemideae) at a continental crossroads: molecular insights into migrations, disjunctions, and reticulations among Old and New World species from a Beringian perspective. Mol Phylogenet Evol. 2012;64(3):471–90. 22580463 10.1016/j.ympev.2012.05.003

[49] Mas de Xaxars G, Garnatje T, Pellicer J, Siljak-Yakovlev S, Vallès J, Garcia S. Impact of dysploidy and polyploidy on the diversification of high mountain *Artemisia* (Asteraceae) and allies. Alp Bot. 2016;126(1):35–48.

[50] Tutin TG. *Artemisia* L. In: Tutin TG, Heywood VH, Burges NA, Moore DM, Valentine DH, Walters SM, et al., editors. Flora Europaea, vol. 4. Cambridge: Cambridge University Press; 1976. p. 178–86.

[51] Yamashiro T, Ogawa M, Yamashiro A, Maki M. Natural hybridization between the endangered herb *Artemisia gilvescens* (Asteraceae) and the common congener, *Artemisia indica* var. *maximowiczii* in Japan. Acta Phytotax Geobot. 2018;69(2):109–17.

