# Supplementary material for: Taming the giant: towards a global sectional taxonomy for the big genus Artemisia
Source: BMC Plant Biol. 2026 May 28;26:1276. doi: 10.1186/s12870-026-08951-7 (PMC13412308; doi:10.1186/s12870-026-08951-7)
Supplement: Supplementary file 2 — Supplementary Material 2. Taxonomic inventory of Artemisia species, detailing their subgeneric and sectional placements. [file 12870_2026_8951_MOESM2_ESM.docx]

**Supplementary Material 2. Taxonomic inventory of *Artemisia* species, detailing their subgeneric and sectional placements.**

1 ***Artemisia*** subg. ***Dracunculus*** (Besser) Rydb.

1.1 ***Artemisia*** sect. ***Palustris*** B.H.Jiao & T.G.Gao

**Species included (2)**: *Artemisia aurata* Kom., *A.* *palustris* L.

1.2 ***Artemisia*** sect. ***Salsoloides*** Leonova.

**Species included (3)**: *Artemisia brachanthemoides* C.Winkl., *A. rubiginosa* B.H.Jiao & T.G.Gao, *A. salsoloides* Willd.

1.3 ***Artemisia*** sect. ***Subarcticae*** B.H.Jiao & T.G.Gao

**Species included (5)**: *Artemisia bejdemaniae* Leonova, *A. norvegica* Fr., *A. punctigera* Krasch. ex Poljakov, *A. sinanensis* Y.Yabe, *A.* *subarctica* Krasch.

1.4 ***Artemisia*** sect. ***Laciniatae*** (Kitam.) B.H.Jiao & T.G.Gao

**Species included (16)**: *Artemisia arctisibirica* Korobkov, *A. macrorhiza* Turcz., *A. remotiloba* Krasch. ex Poljakov, *A. transbaicalensis* Leonova, *A. armeniaca* Lam., *A. atrata* Lam., *A. insipida* Vill., *A. laciniata* Willd., *A. latifolia* Ledeb., *A. maximovicziana* Krasch. ex Poljakov, *A. medioxima* Krasch. ex Poljakov, *A. oelandica* (Besser) Krasch., *A. pancicii* Ronniger ex Danihelka & Marhold, *A. phaeolepis* Krasch., *A. sibirica* (L.) Maxim., *A. tanacetifolia* L.

1.5 ***Artemisia*** sect. ***Glochistigma*** (Kitam.) B.H.Jiao & T.G.Gao

**Species included (2)**: *Artemisia keiskeana* Miq., *A. pedunculosa* Miq.

1.6 ***Artemisia*** sect. ***Dracunculus*** Besser

**Species included (11)**: *Artemisia daghestanica* Krasch. & Poretzky, *A. dimoana* Popov, *A. dracunculiformis* Krasch., *A. dracunculus* L., *A. dubia* Wall. ex Besser, *A. giraldii* Pamp., *A. glauca* Pall. ex Willd., *A. kotuchovii* Kupr., *A. nanschanica* Krasch., *A. tridactyla* Hand.-Mazz., *A. waltonii* J.R.Drumm. ex Pamp.

1.7 ***Artemisia*** sect. ***Latilobus*** Y.R.Ling

**Species included (82)**: *Artemisia abolinii* Lazkov, *A. albicerata* Krasch., *A. borealis* Pall., *A. chienshanica* Ling & W.Wang, *A. congesta* Kitam., *A. gyangzeensis* Ling & Y.R.Ling, *A. hallaisanensis* Nakai, *A. henriettae* Krasch., *A. karavajevii* Leonova, *A. lipskyi* Poljakov, *A. nortonii* Pamp., *A. olgensis* (Vorobiev) Vorosch., *A. oligocarpa* Hayata, *A. pannosa* Krasch., *A. pengchuoensis* Y.R.Ling & S.Y.Zhao, *A. pewzowii* Krasch., *A. quinqueloba* Trautv., *A. remosa* Sugaw., *A. richardsoniana* Besser, *A. stricta* Edgew., *A. swatensis* Podlech, *A. trautvetteriana* Besser, *A. tschernieviana* Besser, *A. angustissima* Nakai, *A. arenaria* DC., *A. bargusinensis* Spreng., *A. campestris* L., *A. cashemirica* M.K.Kaul & S.K.Bakshi, *A. capillaris* Thunb., *A. crithmifolia* L.,*A. demissa* Krasch., *A. depauperata* Krasch., *A. desertorum* Spreng., *A. dolosa* Krasch., *A. duthreuil-de-rhinsii* Krasch., *A. eranthema* Bunge, *A. eriocarpa* Bunge, *A. eriopoda* Bunge, *A. flahaultii* Emb. & Maire, *A. forrestii* W.W.Sm., *A. gansuensis* Ling & Y.R.Ling, *A. globosa* Krasch., *A. globosoides* Ling & Y.R.Ling, *A. halodendron* Turcz. ex Besser, *A. hancei* (Pamp.) Ling & Y.R.Ling, *A. implicata* Leonova, *A. insularis* Kitam., *A. japonica* Thunb., *A. jordanica* Danin, *A. kelleri* Krasch., *A. klementzae* Krasch., *A. kuschakewiczii* C.Winkl., *A. ledebouriana* Besser, *A. limosa* Koidz., *A. littoricola* Kitam., *A. longipetiolata* Charit., *A. macilenta* (Maxim.) Krasch., *A. mairei* H.Lév., *A. manshurica* (Kom.) Kom., *A. marschalliana* Spreng., *A. monosperma* Delile, *A. morrisonensis* Hayata, *A. mustangensis* Yonek., *A. niitakayamensis* Hayata, *A. ordosica* Krasch., *A. oxycephala* Kitag., *A. parviflora* Roxb. ex D.Don, *A.* *pewzowi* C.Winkl., *A. prattii* (Pamp.) Ling & Y.R.Ling, *A. pubescens* Ledeb., *A. pycnocephala* DC., *A. pycnorrhiza* Ledeb., *A. saposhnikovii* Krasch. ex Poljakov, *A. scoparia* Waldst. & Kit., *A. songarica* Schrenk, *A. sphaerocephala* Krasch., *A. tomentella* Trautv., *A. tsugitakaensis* (Kitam.) Ling & Y.R.Ling, *A. wellbyi* Hemsl. & H.Pearson, *A. wudanica* Liou & W.Wang, *A. xanthochroa* Krasch., *A. xigazeensis* Y.R.Ling & M.G.Gilbert, *A. yamadae* (Kitam.) Hideki Takah. & Barkalov.

2 ***Artemisia*** subg. ***Pectinatae*** B.H.Jiao & T.G.Gao

2.1 ***Artemisia*** sect. ***Pectinatae*** B.H.Jiao & T.G.Gao

**Species included (6)**: *Artemisia abyssinica* Sch.Bip. ex Oliv. & Hiern, *A. baxoiensis* B.H.Jiao & T.G.Gao, *A. klotzschiana* Besser, *A. pectinata* Pall, *A. schimperi* Sch.Bip. ex Engl., *A. tilhoana* Quézel.

2.2 ***Artemisia*** sect. ***Hedinianae*** (Y.R.Ling) B.H.Jiao & T.G.Gao

**Species included (4)**: *Artemisia biennis* Willd., *A. hedinii* Ostenf., *A. magellanica* Sch.Bip., *A. tournefortiana* Rchb.

3 ***Artemisia*** subg. ***Pacifica*** C.R.Hobbs & B.G.Baldwin

3.1 ***Artemisia*** sect. ***Pacifica*** (C.R.Hobbs & B.G.Baldwin) B.H.Jiao & T.G.Gao

**Species included (4)**: *Artemisia australis* Less., *A. chinensis* L., *A. kauaiensis* (Skottsb.) Skottsb., *A. mauiensis* Skottsb.

4 ***Artemisia*** subg. ***Ponticae*** (Rydb.) B.H.Jiao & T.G.Gao

4.1 ***Artemisia*** sect. ***Ponticae*** (Rydb.) B.H.Jiao & T.G.Gao

**Species included (23)**: *Artemisia abrotanum* L., *A. adamsii* Besser, *A. andersiana* Podlech, *A. afra* Jacq. ex Willd., *A. aksaiensis* Y.R.Ling, *A. alba* Turra, *A. baimaensis* Y.R.Ling & Z.C.Chuo, *A. brachyloba* Franch., *A. chamaemelifolia* Vill., *A. dalai-lamae* Krasch., *A. freyniana* (Pamp.) Krasch., *A. gmelinii* Weber ex Stechm., *A. hololeuca* M.Bieb. ex Besser, *A. macrantha* Ledeb., *A. mesatlantica* Maire, *A. molinieri* Quézel, Barbero & R.J.Loisel, *A. negrei* A.Ouyahya, *A. olchonensis* Leonova, *A. persica* Boiss., *A. polybotryoidea* Y.R.Ling, *A. pontica* L., *A. stechmanniana* Besser, *A. vestita* Wall. ex Besser.

5 ***Artemisia*** subg. ***Seriphidium*** Besser ex Less.

5.1 ***Artemisia*** sect. ***Annuae*** (Rydb.) B.H.Jiao & T.G.Gao

**Species included (3)**: *Artemisia annua* L., *A. caruifolia* Buch.-Ham. ex Roxb., *A. calcicola* X.Q.Guo & L.Wang.

5.2 ***Artemisia*** sect. ***Anethifoliae*** (Poljakov) B.H.Jiao & T.G.Gao

**Species included (6)**: *Artemisia anethifolia* Weber ex Stechm., *A. anethoides* Mattf., *A. fauriei* Nakai, *A. fukudo* Makino, *A. nakaii* Pamp., *A. przewalskii* Krasch.

5.3 ***Artemisia*** sect. ***Seriphidium*** (Besser ex Less.) Hooker

**Species included (119):** *Artemisia aflatunensis* Poljakov ex U.P.Pratov & Bakanova, *A. albicaulis* Nevski, *A. algeriensis* Filatova, *A. amoena* Poljakov, *A. aralensis* Krasch., *A. araxina* Takht., *A. arenicola* Krasch. ex Poljakov, *A. assurgens* Filatova, *A. aucheri* Boiss., *A. badghysi* Krasch. & Lincz. ex Poljakov, *A. balchanorum* Krasch., *A. baldshuanica* Krasch. & Zaprjag., *A. barrelieri* Besser, *A. bashkalensis* Kursat & Civelek, *A. bicolor* Rech.f. & Wagenitz, *A. borotalensis* Poljakov, *A. brevifolia* Wall. ex DC., *A. caerulescens* L., *A. camelorum* Krasch., *A. chitralensis* Podlech, *A. cina* O.Berg, *A. ciniformis* Krasch. & Popov ex Poljakov, *A. compacta* Fisch. ex DC., *A. czukavinae* Filatova, *A. densiflora* Viv., *A. densifolia* Filatova, *A. diffusa* Krasch. ex Poljakov, *A. dubjanskyana* Krasch. ex Poljakov, *A. dumosa* Poljakov, *A. dzevanovskyi* Leonova, *A. elongata* Filatova & Ladygina, *A. eremophila* Krasch. & Butkov ex Poljakov, *A. fedorovii* Rzazade, *A. fedtschenkoana* Krasch., *A. ferganensis* Krasch. ex Poljakov, *A. finita* Kitag., *A. fragrans* Willd., *A. freitagii* Podlech, *A. fulvella* Filatova & Ladygina, *A. ghazniensis* Podlech, *A. ghoratensis* Podlech, *A. glanduligera* Krasch. ex Poljakov, *A. glaucina* Krasch. ex Poljakov, *A. gracilescens* Krasch. & Iljin, *A. grenardii* Franch., *A. gurganica* (Krasch.) Filatova, *A. gypsacea* Krasch., Popov & Lincz. ex Poljakov, *A. halophila* Krasch., *A. heptapotamica* Poljakov, *A. herba-alba* Asso, *A. huguetii* Caball., *A. ifranensis* J.Didier, *A. inculta* Sieber ex DC., *A. issykkulensis* Poljakov, *A. kandaharensis* Podlech, *A. karatavica* Krasch. & Abolin ex Poljakov, *A. kasakorum* (Krasch.) Pavlov, *A. kaschgarica* Krasch., *A. kemrudica* Krasch., *A. kermanensis* Podlech, *A. knorringiana* Krasch., *A. kochiiformis* Krasch. & Lincz. ex Poljakov, *A. kopetdaghensis* Krasch., Popov & Lincz. ex Poljakov, *A. korovinii* Poljakov, *A. korshinskyi* Krasch. ex Poljakov, *A. kurramensis* Qazilb., *A. lehmanniana* Bunge, *A. lercheana* Weber ex Stechm., *A. lessingiana* Besser, *A. leucotricha* Krasch. ex Ladygina, *A. maritima* L., *A. minchunensis* (Y.R.Ling) Long Wang & G.Z.Jin, *A. mogoltavica* Poljakov, *A. mongolorum* Krasch., *A. mucronulata* Poljakov, *A. namanganica* Poljakov, *A. nigricans* Filatova & Ladygina, *A. nitrosa* Weber ex Stechm., *A. nutans* Willd., *A. oliveriana* J.Gay ex Besser, *A. oranensis* Deb. ex Filatova, *A. pauciflora* Weber ex Stechmann, *A. pineticola* Kupr., *A. pineticola* Kupr., *A. porrecta* Krasch. ex Poljakov, *A. prolixa* Krasch. ex Poljakov, *A. qingheensis* G.Z.Jin, *A. quettensis* Podlech, *A. ramosa* C.Sm. ex Link, *A. rhodantha* Rupr., *A. saharae* Pomel, *A. saissanica* (Krasch.) Filatova, *A. santolina* Schrenk, *A. santonicum* L., *A. sawanensis* (Y.R.Ling & Humphries) Long Wang & G.Z.Jin, *A. schrenkiana* Ledeb., *A. scopiformis* Ledeb., *A. scotina* Nevski, *A. semiarida* (Krasch. & Lavrenko) Filatova, *A. sieberi* Besser, *A. spicigera* K.Koch, *A. stenocephala* Krasch. ex Poljakov, *A. subchrysolepis* Filatova, *A. sublessingiana* Krasch. ex Poljakov, *A. subsalsa* Filatova, *A. szowitziana* (Besser) Grossh., *A. taurica* Willd., *A. tecti-mundi* Podlech, *A. tenuisecta* Nevski, *A. terrae-albae* Krasch., *A. thomsoniana* (C.B.Clarke) Filatova, *A. tianschanica* Krasch. ex Poljakov, *A. transiliensis* Poljakov, *A. turanica* Krasch., *A. turcomanica* Gand., *A. vachanica* Krasch. ex Poljakov, *A. valida* Krasch. ex Poljakov, *A. Vallèsiaca* All.

6 ***Artemisia*** subg. ***Tridentatae*** (Rydb.) McArthur

6.1 ***Artemisia*** sect. ***Younghusbandianae*** B.H.Jiao & T.G.Gao

**Species included (1)**: *Artemisia younghusbandii* J.R.Drumm. ex Pamp.

6.2 ***Artemisia*** sect. ***Lagocephalae*** (Kitam.) B.H.Jiao & T.G.Gao

**Species included (3)**: *Artemisia kruhsiana* Besser, *A. lagocephala* (Fisch. ex Besser) DC., *A. rutifolia* Stephan ex Spreng.

6.3 ***Artemisia*** sect. ***Tridentatae*** L.M.Shultz

**Species included (36)**: *Artemisia alaskana* Rydb., *A. albicans* Sòn.Garcia, Garnatje, McArthur, Pellicer, S.C.Sand. & Vallès-Xirau, *A. arbuscula* Nutt., *A. bigelovii* A.Gray, *A. californica* Less., *A. cana* Pursh, *A. capitata* (Nutt.) Sòn.Garcia, Garnatje, McArthur, Pellicer, S.C.Sand. & Vallès-Xirau, *A. constricta* Sòn.Garcia, Garnatje, McArthur, Pellicer, S.C.Sand. & Vallès-Xirau, *A. copa* Phil., *A. echegarayi* Hieron., *A. filifolia* Torr., *A. franserioides* Greene, *A. furcata* M.Bieb., *A. globularia* Cham. ex Besser, *A. inaequifolia* Sòn.Garcia, Garnatje, McArthur, Pellicer, S.C.Sand. & Vallès-Xirau, *A. martirensis* (Wiggins) C.R.Hobbs & B.G.Baldwin, *A. mendozana* DC., *A. nesiotica* P.H.Raven, *A. nova* A.Nelson, *A. nuttallii* (Torr. & A.Gray) Mosyakin, L.M.Shultz & G.V.Boiko, *A. papposa* S.F.Blake & Cronquist, *A. pattersonii* A.Gray, *A. pedatifida* Nutt., *A. porteri* Cronquist, *A. potentilloides* A.Gray, *A. pygmaea* A.Gray, *A. rigida* (Nutt.) A.Gray, *A. rothrockii* A.Gray, *A. ruthiae* (A.H.Holmgren, L.M.Shultz & Lowrey) Sòn.Garcia, Garnatje, McArthur, Pellicer, S.C.Sand. & Vallès-Xirau, *A. scopulorum* A.Gray, *A. simplex* (A.Nelson) Sòn.Garcia, Garnatje, McArthur, Pellicer, S.C.Sand. & Vallès-Xirau, *A. sodiroi* Hieron., *A. spiciformis* Osterh., *A. spinescens* D.C.Eaton, *A. tridentata* Nutt., *A. tripartita* Rydb.

7 ***Artemisia*** subg. ***Absinthium*** (Miller) Lessing

7.1 ***Artemisia*** sect. ***Blepharolepides*** (Y.R.Ling) B.H.Jiao & T.G.Gao

**Species included (1)**: *Artemisia blepharolepis* Bunge.

7.2 ***Artemisia*** sect. ***Sieversianae*** (Kitam.) B.H.Jiao & T.G.Gao

**Species included (7)**: *Artemisia jacutica* Drobow, *A. macrocephala* Jacquem. ex Besser, *A. pallens* Wall. ex DC., *A. samoiedorum* Pamp., *A. shangnanensis* Ling & Y.R.Ling, *A. sieversiana* Ehrh. ex Willd., *A. succulenta* Ledeb.

7.3 ***Artemisia*** sect. ***Absinthium*** (Mill.) DC.

**Species included (5)**: *Artemisia absinthium* L., *A. arborescens* L., *A. argentea* L'Hér., *A. gorgonum* Webb, *A. thuscula* Cav.

7.4 ***Artemisia*** sect. ***Junceae*** Poljakov ex Filatova

**Species included (4)**: *Artemisia deserti* Krasch., *A. juncea* Kar. & Kir., *A. leucodes* Schrenk, *A. macrosciadia* Poljakov.

7.5 ***Artemisia*** sect. ***Frigidae*** (Rydb.) B.H.Jiao & T.G.Gao

**Species included (48)**: *Artemisia abbreviata* (Krasch. ex Korobkov) Krasnob., *A. aleutica* Hultén, *A. alpina* Pall. ex Willd., *A. argyrophylla* Ledeb., *A. aschurbajewi* C.Winkl., *A. assoana* Willk., *A. atlantica* Coss. & Durieu, *A. austriaca* Jacq., *A. caespitosa* Ledeb., *A. cuspidata* Krasch., *A. czekanowskiana* Trautv., *A. davazamczii* Darijma & Kamelin, *A. disjuncta* Krasch., *A. eriantha* Ten., *A. filatovae* Kupr., *A. frigida* Willd., *A. genipi* Stechm., *A. glacialis* L., *A. glomerata* Ledeb., *A. granatensis* Boiss., *A. haussknechtii* Boiss., *A. hippolyti* A.Butkov, *A. incana* Druce, *A. judaica* L., *A. kitadakensis* Hara & Kitam., *A. lagopus* Fisch. ex Besser, *A. aethiopica* L., *A. melanolepis* Boiss., *A. minor* Jacquem. ex Besser, *A. nepalensis* Nees, *A. nitida* Bertol., *A. nivalis* Braun-Blanq., *A. obtusiloba* Ledeb., *A. pedemontana* Balb., *A. penicilliformis* (Shih) M.Wei & T.G.Gao, *A. radicans* Kupr., *A. reptans* C.Sm., *A. rupestris* L., *A. schmidtiana* Maxim., *A. senjavinensis* Besser, *A. sericea* Weber ex Stechm., *A. skorniakovii* C.Winkl., *A. splendens* Willd., *A. succulentoides* Ling & Y.R.Ling, *A. umbelliformis* Lam., *A. viridis* Willd. ex DC., *A. woodii* (Neilson) C.W.Riggins, *A. xerophytica* Krasch.

8 ***Artemisia*** subg. ***Artemisia***

8.1 ***Artemisia*** sect. ***Selengenses*** (Pamp.) B.H.Jiao & T.G.Gao

**Species included (14)**: *Artemisia amygdalina* Decne., *A. anomala* S.Moore, *A. carruthii* Alph.Wood ex J.H.Carruth, *A. deversa* Diels, *A. douglasiana* Besser, *A. longifolia* Nutt., *A. ludoviciana* Nutt., *A. michauxiana* Besser, *A. packardiae* J.W.Grimes & Ertter, *A. selengensis* Turcz. ex Besser, *A. serrata* Nutt., *A. suksdorfii* Piper, *A. tilesii* Ledeb., *A. viridissima* Pamp.

8.2 ***Artemisia*** sect. ***Artemisia***

**Species included (97)**: *Artemisia abaensis* Y.R.Ling & S.Y.Zhao, *A. argyi* H.Lév. & Vaniot, *A. atrovirens* Hand.-Mazz., *A. austrohimalayaensis* Y.R.Ling & Puri, *A. austroyunnanensis* Ling & Y.R.Ling, *A. banihalensis* M.K.Kaul & S.K.Bakshi, *A. bhutanica* Grierson & Spring., *A. brachyphylla* Kitam., *A. calophylla* Pamp., *A. campbellii* Hook.f. & Thomson ex C.B.Clarke, *A. chingii* Pamp., *A. codringtonii* Rech.f., *A. comaiensis* Ling & Y.R.Ling, *A. divaricata* (Pamp.) Pamp., *A. domingensis* Urb., *A. emeiensis* Y.R.Ling, *A. eriocephala* Pamp., *A. erlangshanensis* Y. Ling & Y. R. Ling, *A. estesii* K.L.Chambers, *A. flaccida* Hand.-Mazz., *A. fulgens* Pamp., *A. gilvescens* Miq., *A. gongshanensis* Y.R.Ling & Humphries, *A. gyitangensis* Ling & Y.R.Ling, *A. hanwulaensis* Y.Z.Zhao, *A. igniaria* Maxim., *A. imponens* Pamp., *A. incisa* Pamp., *A. indica* Willd., *A. integrifolia* L., *A. javanica* Pamp., *A. jilongensis* Y.R.Ling & Humphries, *A. kanashiroi* Kitam., *A. kawakamii* Hayata, *A. koidzumii* Nakai, *A. lactiflora* Wall. ex DC., *A. lanaticapitula* X.F.Jin, Z. H. Chen & Y.F.Lu, *A. lancea* Vaniot, *A. leucophylla* C.B.Clarke, *A. lingyeouruennii* L.M.Shultz & Boufford, *A. mattfeldii* Pamp., *A. mongolica* (Fisch. ex Besser) Nakai, *A. monophylla* Kitam., *A. montana* (Nakai) Pamp., *A. moorcroftiana* Wall. ex DC., *A. myriantha* Wall. ex Besser, *A. nepalica* Yonek., *A. nilagirica* (C.B.Clarke) Pamp., *A. nujianensis* (Ling & Y.R.Ling) Y.R.Ling, *A. occidentalisichuanensis* Y.R.Ling & S.Y.Zhao, *A. occidentalisinensis* Y.R.Ling, *A. orientalihengduangensis* Ling & Y.R.Ling, *A. orientalixizangensis* Y.R.Ling & Humphries, *A. orientaliyunnanensis* Y.R.Ling, *A. palmeri* A.Gray, *A. phyllobotrys* (Hand.-Mazz.) Ling & Y.R.Ling, *A. princeps* Pamp., *A. pringlei* Greenm., *A. qinlingensis* Ling & Y.R.Ling, *A. robusta* (Pamp.) Ling & Y.R.Ling, *A. rosthornii* Pamp., *A. roxburghiana* Besser, *A. rubripes* Nakai, *A. saitoana* Kitam., *A. shennongjiaensis* Ling & Y.R.Ling, *A. sichuanensis* Ling & Y.R.Ling, *A. simulans* Pamp., *A. neosinensis* B.H.Jiao & T.G.Gao, *A. smithii* Mattf., *A. somae* Hayata, *A. speciosa* (Pamp.) Ling & Y.R.Ling, *A. stelleriana* Besser, *A. stipularis* Urb. & Ekman, *A. stolonifera* (Maxim.) Kom., *A. subulata* Nakai, *A. sylvatica* Maxim., *A. tafelii* Mattf., *A. tainingensis* Hand.-Mazz., *A. tangutica* Pamp., *A. thellungiana* Pamp., *A. tsuneoi* Tatew. & Kitam., *A. tukuchaensis* Kitam., *A. umbrosa* (Besser) Turcz. ex Verl., *A. velutina* Pamp., *A. verbenacea* (Kom.) Kitag., *A. verlotiorum* Lamotte, *A. vexans* Pamp., *A. viridisquama* Kitam., *A. viscida* Pamp., *A. viscidissima* Ling & Y.R.Ling, *A. vulgaris* L., *A. yadongensis* Ling & Y.R.Ling, *A. yongii* Y.R.Ling, *A. yunnanensis* Jeffrey, *A. zayuensis* Ling & Y.R.Ling, *A. zhongdianensis* Y.R.Ling.
